# Improving Fungal Cultivability for Natural Products Discovery

**DOI:** 10.3389/fmicb.2021.706044

**Published:** 2021-09-16

**Authors:** Teppo Rämä, C. Alisha Quandt

**Affiliations:** ^1^Marbio, Norwegian College of Fishery Science, University of Tromsø – The Arctic University of Norway, Tromsø, Norway; ^2^Department of Ecology and Evolutionary Biology, University of Colorado, Boulder, Boulder, CO, United States

**Keywords:** fungi, cultivation, growth factors, spore germination, filamentous growth, omics, biodiscovery, *in situ* culturing

## Abstract

The pool of fungal secondary metabolites can be extended by activating silent gene clusters of cultured strains or by using sensitive biological assays that detect metabolites missed by analytical methods. Alternatively, or in parallel with the first approach, one can increase the diversity of existing culture collections to improve the access to new natural products. This review focuses on the latter approach of screening previously uncultured fungi for chemodiversity. Both strategies have been practiced since the early days of fungal biodiscovery, yet relatively little has been done to overcome the challenge of cultivability of as-yet-uncultivated fungi. Whereas earlier cultivability studies using media formulations and biological assays to scrutinize fungal growth and associated factors were actively conducted, the application of modern omics methods remains limited to test how to culture the fungal dark matter and recalcitrant groups of described fungi. This review discusses the development of techniques to increase the cultivability of filamentous fungi that include culture media formulations and the utilization of known chemical growth factors, *in situ* culturing and current synthetic biology approaches that build upon knowledge from sequenced genomes. We list more than 100 growth factors, i.e., molecules, biological or physical factors that have been demonstrated to induce spore germination as well as tens of inducers of mycelial growth. We review culturing conditions that can be successfully manipulated for growth of fungi and visit recent information from omics methods to discuss the metabolic basis of cultivability. Earlier work has demonstrated the power of co-culturing fungi with their host, other microorganisms or their exudates to increase their cultivability. Co-culturing of two or more organisms is also a strategy used today for increasing cultivability. However, fungi possess an increased risk for cross-contaminations between isolates in existing *in situ* or microfluidics culturing devices. Technological improvements for culturing fungi are discussed in the review. We emphasize that improving the cultivability of fungi remains a relevant strategy in drug discovery and underline the importance of ecological and taxonomic knowledge in culture-dependent drug discovery. Combining traditional and omics techniques such as single cell or metagenome sequencing opens up a new era in the study of growth factors of hundreds of thousands of fungal species with high drug discovery potential.

## Introduction

The natural products discovery (NPD) community has long been aware of microbial interactions as the driver for chemical diversity. The interactions in nature are complex involving interplay between tens or hundreds of microbes, plants, animals, and the abiotic environment, of which the taxon-taxon interactions seem to be more important (Lima-Mendez et al., 2015). Hypotheses on species interactions are difficult to test in such a complex system. We are forced to simplify the systems under controlled laboratory environments for simulating microbial interactions and their associated chemical compounds. Consequently, cultured organisms are needed.

Microbial cultures are crucial because of the huge amounts of molecular data on microbes’ identities, biosynthetic gene clusters (BGCs), and produced metabolites or proteins are accumulating at an increasing pace. These data demand experiments to interpret their meaning. In addition to their traditional importance for isolating new diversity and producing enough fresh biomass to be used for NPD (i.e., for pure compound isolation, structure elucidation, and bioactivity testing), the new roles of cultures have become evident subsequent to the development of omics techniques. Omics data are valuable for creating ecological and physiological hypotheses, but cultures are at least equally valuable, as they are needed to test the hypotheses using single isolates, co-cultures of microbial pairs or consortia, and the compounds produced from the organisms. Moreover, characterizing and catalogizing genes and gene products from cultured microorganisms is crucial for improving reference databases that omics tools rely on to annotate and interpret chemical or biological data from any study system.

The molecular databases and their systematic development are an evolving framework from which we can place novel sequences, genes, gene clusters, and gene products discovered using metagenomics (new species), metatranscriptomics (new genes), metabolomics (new compounds), or metaproteomics (new proteins). Accumulation of these data and their decodification generates a positive feedback loop of information between culturing and culture-independent studies. It is apparent that microbial cultures are needed more than ever for NPD and other fields of microbiology.

### Successes and Challenges in Industrial Production and Application of NPs From Uncultured Fungi

The fungal collections around the world host voucher specimens of more than a hundred thousand species of fungi and obviously tens of thousands of these described species remain uncultured, or are recalcitrant to culture. Lack of authentic strains hinders NPD, and especially industrial-scale production of some well-known potent fungal NPs and their derivatives. However, several success stories of fungal NPD from, at-the-time uncultured fungi have been documented, yet further development of culturing protocols would likely result into wider commercial application and more opportunities for success. Here, we recount three examples (in *Amanita*, Clavicipitaceae, and lichenized fungi) of the potential, success, and challenges of NPD in uncultured fungi.

Some of the most iconic fungi, such as common *Amanita* (Agaricales, Basidiomycota) species, are hard to culture. These are large fruiting body forming mushrooms that unfortunately often end up consumed by humans with fatal consequences. Species in the genus *Amanita* are mostly ectomycorrhizal fungi that form associations with many types of trees. *Amanita phalloides, A. bisporigera, and A. verna* among others produce amatoxins that are some of the most deadly NPs known. These are ribosomally synthesized and post-translationally modified peptides (RiPPS) that can also be found in some saprotrophic Agaricomycetes fungi, such as *Galerina* and *Lepiota*, but in significantly lower titers (Pahl et al., 2019). Chemical synthesis is currently the most promising way of amatoxin production (Lutz et al., 2020). However, finding a culturing protocol suitable for axenic culturing of the most prolific amatoxin producers on synthetic media may provide a more economic strategy to improve the limited access to these compounds that are extensively used in cell biology to study RNA polymerase II and mRNA synthesis and represent ideal payloads for antibody-drug conjugates (Pahl et al., 2019).

Ergot alkaloid producing ascomycetes are an example of fungi where culturing improvements have proven crucial for industrial production to meet societal needs, and where further culturing innovations would be needed to better access the full chemical arsenal of these fungi. Clavicipitiaceae fungi in the phylum Ascomycota are endophytes found in several plant families. These fungi are known for the production of biotechnologically relevant chemistry including ergot alkaloids that are used as medicinal agents and are present as toxins in the environment causing toxification of livestock feeding on the plants containing the alkaloids (Kelleher, 1969). The most famous alkaloid producer is the fungus *Claviceps purpurea*, also known as Saint Anthony’s fire and Ergot of Rye, that is responsible for tens of thousands of people falling ill under ergotism disease since the Middle Ages and has also been associated with witch-hunts of the past (Caporael, 1976; Matossian, 1982). The source of hallucinations, abnormal behavior, and self-recognitions of people convicted and burned as witches and severe poisonings even deaths seen under ergotism epidemics was infested cereals, especially rye, consumed by human populations. The societal importance of *C. purpurea* contributed to the early use of its alkaloids in labor induction since 1582, whereas the medicinal introduction took place in the early nineteenth century, many decades before *C. purpurea* was cultured (Bonns, 1922; Kelleher, 1969; Meyer, 1888; Brefeld, 1891). The industrial utilization of ergot alkaloids was based on extracts prepared from field-collected sclerotia that were of high value and culturing studies were later initiated out of “pressure from the economic conditions” (Bonns, 1922). It took decades of empirical and experimental work to develop these fungi for industrial fermentation.

*Claviceps purpurea*, as well as some other clavicipiticeous fungi can be cultured and their chemical products harvested for commercial utilization, but for most species we do not know how to culture them on synthetic media (Keller and Tudzynski, 2002). Some of the as-yet-uncultured Clavicipitiaceae fungi include species of the genus *Periglandula* with two described (*P. ipomoeae* and *P. turbinae*) and several undescribed species that are symbionts of dicot plants in the Convolvulaceae family (Steiner et al., 2011; Beaulieu et al., 2015). The two *Periglandula* species produce potent mycotoxins that protect host plants (*Ipomoea asarifolia* and *Turbina corymbosa*) against herbivores. The ergot alkaloids concentrations in seeds of related plants in Convolvulaceae exceed the concentrations found in Clavicipitiaceae-infected grasses up to 1,000-fold (Beaulieu et al., 2013). Altogether 40 Convolvulaceae plant species are reported to contain ergot alkaloids (Eich, 2008) and it has been known for more than a half century that these alkaloids are similar to the ones found in clavicipitaceous fungi (Schultes, 1941, 1969). However, it was only recently discovered that *Periglandula* species are the real producers of the ergot alkaloids detected in Convolvulaceae (Kucht et al., 2004; Steiner et al., 2006; Schardl et al., 2013). The full metabolic capacity of the described *Periglandula* and the recently discovered species remain understudied and may include novel chemodiversity with biomedical relevance that is currently hard to study due to the uncultivability of the strains.

The chemical synthesis of ergot alkaloids needs more efficient strategies, and synthetic biology including heterologous expression has not yielded good, economical solutions for the production of ergot alkaloids (Keller and Tudzynski, 2002; Chen et al., 2017; Liu and Jia, 2017). The industrial production relies on liquid culturing of selected Clavicipiaceae strains that are unstable and prone to degeneration during culturing and preservation, or parasitic cultures that are sensitive to changing climatic conditions and suitable production locations that allow Clavicipitiaceae to be cultured in rye despite the proximity of other cereals harvested for human consumption, or inefficient semisynthetic approaches for the production of several types of ergot alkaloids. Developments are needed for a versatile and economic production of ergot alkaloids and the promising potential of *Periglandula* species as high-concentration ergot alkaloid producers is an important hypothesis to test, but for step one, we need to find ways to culture these fungi.

Lichens are also a good source of diverse NPs with biological activities and medicinal relevance including aliphatic acids, pulvinic acid derivatives, depsides and depsidones, dibenzofurans, anthraquinones, naphthoquinones, and epidithiopiperazinediones some of which are of considerable commercial interest (Müller, 2001). Lichens are by nature slow growing and the relatively small thalli in the field provide a limited and often non-sustainable source for NPD. Culturing of lichens on synthetic medium is in many cases impossible as culture conditions should sustain a complex biological system in a reproducible manner consisting of a photobiont, mycobiont, and in some cases also associated cyanobacterium, other fungi or non-photo-autotrophic bacteria (Hodkinson et al., 2012; Spribille et al., 2016). However, these symbiotic associations produce mostly unique natural products that are seldom found in non-lichenized fungi or photosynthetic organisms (Müller, 2001).

One way to overcome the recalcitrant growth of lichens in culture is to establish pure cultures of the mycobiont that often shows an increased growth in culture compared to the symbiotic association. Aposymbiotic cultures of lichen mycobionts can be established using polyspore colonies from discharged sexual ascospores that disseminate the fungal partner (Hamada, 1989). Screening aposymbiotic mycobiont cultures for bioactive NPs is a relatively recent strategy in NPD that has produced promising results and shows that lichens can produce many kinds of NPs depending on which dimension of the symbiotic association is targeted. Multiple studies have screened aposymbiotic mycobiont cultures for NPs with exciting results that highlight that mycobionts can produce novel NPs in axenic cultures that cannot be found in free living lichen thalli. For example, Fazio et al. (2018) cultured the mycobiont of *Graphis elongata* lichen separately from its photobiont that resulted in the production of the rare elsinochrome A pigment. The production of this compound is likely silenced in the symbiotic association due to the toxicity of the pigment to the photobiont. Takenaka et al. (2011) fermented mycobionts of *G. proserpens* for 11 months and reports six new compounds that have not been found in lichen thalli before.

An alternative strategy to aposymbiotic cultures of mycobiont produced lichen compounds is to use mass-spectrometry imaging (MSI) of living lichens. This strategy can be used to detect compounds in a spatial context and co-localize compounds with the mycobiont or the photobiont. Laser desorption/ionization mass spectrometric imaging (LDI-MSI) is the preferred method as it allows spatial mapping of NPs down to 50 um resolution and, in contrast with MALDI-MSI (matrix assisted laser desorption and ionization mass-spectrometry imaging) is not dependent on matrix formation that may interfere with the native distribution of NPs in the studied system (Le Pogam et al., 2016).

Aposymbiotic mycobiont cultures and MSI open up the possibility to study recalcitrantly cultured lichens and especially the NPs produced by lichenized fungi. Aposymbiotic cultures are needed for scale-up production of NPs of interest for NPD. Culturing method developments are needed to provide the mycobiont advantage that allows isolation of non-propagule producing asexual lichens that currently cannot be isolated to aposymbiotic cultures using the method described by Hamada (1989).

The list of societally and commercially important fungal NPs is longer than the examples given above. Studies with *Amanita*, Clavicipitaceae and lichenized fungi show that many fungal NPs could be effectively studied and even industrially utilized before culturing. However, developments of culturing protocols for these specific fungi have or would open up for a wider exploitation of the NPs (or derivatives) as drugs or other biotechnological innovations. In addition to culturing the recalcitrantly cultured described fungi, such as *Amanita* spp., we should find ways to culture more of the undescribed fungi that obviously are more in numbers and more diverse than the fungi named and deposited in fungaria.

### Fungi Are Hyperdiverse and One of the Best Sources for Natural Products

Fungi are heterotrophic eukaryotes that coexist with other microscopic and macroscopic life forms on earth. Symbiotic fungi include mutualists, commensalists, parasites, and pathogens that live in intimate associations with their macroscopic hosts and interacting microbial co-dwellers of any given habitat or substratum. Saprotrophic fungi are often considered to be more independent living in non-intimate associations, but in reality, they seldom (if ever) act alone in nature. Rather, they function as part of an interacting microbial, plant and animal community providing support or suppression of the saprotrophic activity (Woodward and Boddy, 2008).

Fungi are one of the most species rich groups of eukaryotic organisms with estimated 2.2–3.8 million species (Hawksworth and Lücking, 2017). The fungal kingdom currently covers at least nine phyla and conceals several Fungi *incertae sedis* taxa at high taxonomic rank that have poorly understood ecology (Naranjo-Ortiz and Gabaldón, 2019; Li et al., 2021). This richness and diversity of species and the functions of these BGCs supplies a vast pool of fungal secondary metabolites that, in theory, translates to countless opportunities for the biotechnology industry.

**FIGURE 1:**
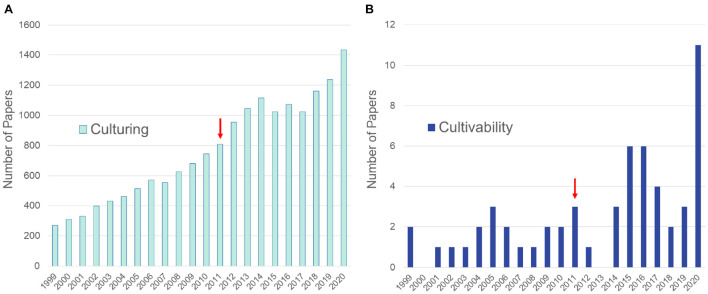
Numbers of articles from the PubMed database queried to show differences in publications referring to culturing of fungi vs. culturability of fungi. **(A)** Results of query (“fungi”[TIAB] OR “fungal”[TIAB]) AND (“culture”[TIAB] OR “culturing”[TIAB] OR “co-culture”[TIAB] OR “co-culturing”[TIAB]). **(B)** Results of query (“fungi”[TIAB] OR “fungal”[TIAB]) AND (“culturability”[TIAB] OR “cultivability”[TIAB]). Arrows denote approximate time frame when high throughput methods became widely used to sequence fungal genomes.

Our access to the fungal secondary metabolites in NPD remains highly limited and their biotechnological potential remains underutilized. Research is increasing reliant on culturing of fungi even with the introduction of omics studies that rapidly have become standard tools in microbiology (Figure 1). However, despite the steady increase of research outputs involving cultured fungi, these studies tend to focus on a limited spectrum of the fungal kingdom, especially certain types of easily cultured yeasts, ascomycetes, basidiomycetes, and zygomycetes. Most relevant for NPD are filamentous ascomycetes and basidiomycetes that are the most prolific producers of NPs, as reflected in the historical record of their products which is mirrored by their larger genome sizes and higher numbers of BGCs per genome encoding for the biosynthesis of secondary metabolites (explore, e.g., genomes at https://mycocosm.jgi.doe.gov/mycocosm/home; Grigoriev et al., 2014). It is a justifiable strategy to target NPD efforts toward fungi of the Dikarya. However, we have less information on the potential of diverse non-dikaryoid fungi to produce NPs, some of which have larger genomes and higher numbers of BGCs, such as *Dimargaris cristalligena* (Mucoromycota) and *Anaeromyces robustus* (Neocallimastigales) (Haitjema et al., 2017; Ahrendt et al., 2018). Many of the BGCs detected in non-dikaryoid fungi are novel and likely to encode for novel NPs, potentially enabling new avenues for biotechnological utilization (see Voigt et al., 2016; Tabima et al., 2020; Koczyk et al., 2021). Numbers and novelty of BGCs in individual taxa of early diverging fungi calls for increasing their cultivability in parallel with cultivability studies on filamentous Dikarya.

Serious investigations focusing on the phenomenon of fungal cultivability represent at the most a small percent of culturing studies during the last decades (Figure 1). Consequently, studies on how to translate previously uncultured strains into metabolic products remain limited. Fungi are currently the most studied and among the most prolific groups of organisms in marine NPD (with altogether 5547 new NPs reported) and can also be found among the top three most prolific sources in the terrestrial environment (Carroll et al., 2020, 2021; Ntie-Kang and Svozil, 2020). However, significant barriers remain to accessing the vast pool of fungal NPs. These include awakening BGCs of the cultured strains that often remain silent during laboratory culturing. Lack of full expression of a fungal strains’s full metabolic potential is an age-old problem and has been discussed extensively (Yarbrough et al., 1993; Dreyfuss and Chapela, 1994; Brakhage and Schroeckh, 2011; Rutledge and Challis, 2015) and will not be treated here. Instead, this review focuses on the approach of culturing previously unstudied fungal diversity and thus gaining access to new NPs.

Several papers have reviewed advances in microbial cultivability, especially bacterial cultivability, highlighting past and recent advances, improvements in culturing methods and introducing major challenges in the field (e.g., Stewart, 2012; Overmann et al., 2017; Lewis et al., 2020). Some of these strategies will be applicable to fungi that have rarely been addressed in more recent cultivability literature. Older work includes several excellent reviews on how to define nutrients and elements essential to fungal growth (Lilly and Barnett, 1951), more specifically growth factors that induce or inhibit the germination of fungal spores (Fries, 1966) or factors that induce the growth of filamentous fungi in culture (Barnett, 1964; Sun and Fries, 1992, and references therein). Whereas this older literature gives historical perspective and practical know how on the major challenges in inducing the growth of fungi decades ago (many challenges still prevailing today), the growth factors for fungi including the recalcitrantly cultured symbiotic species groups have not been revisited for decades. A warmly welcomed exception is the recent review focusing on different microbial cultivation techniques for NPD that also includes relevant studies conducted on fungi (Nai and Meyer, 2018).

We believe that today’s researchers working with fungal NPD will benefit from accumulated knowledge on fungal growth factors published since the early twentieth century. The past research provided the community breakthroughs including characterization of growth factors that can be used to induce fungal growth (e.g., in Lilly and Barnett, 1951), innovative culturing techniques, as well as increased taxonomic and ecological knowledge. Reexamining the old literature and actively applying its knowledge may yield prosperous results today, especially when employed in conjunction with state-of-the-art omics and high-throughput techniques and NP prospecting efforts that extend to a wider geographic area and broader selection of habitats and organism sources than ever.

## Factors Affecting Fungal Growth

Fungi have been cultured for some thousands of years and on solid media in laboratories since the middle of the nineteenth century (Vittadini, 1852; Ainsworth, 1976). Even before the laboratory culturing, field mycologists have made observations about the climatic and ecological conditions that favor growth of different types of fungi in nature (Persoon, 1801; Fries, 1821). These may relate to multiple factors such as annual rainfall or temperature, type of habitat or soil properties or small scale ecophysiological interactions. For example, in the intimate relationship between the bolete *Suillus bovinus* and *Gomphidius roseus* the fruiting bodies of the latter can only be found in the vicinity of fruiting bodies of *S. bovinus* that is forming mycorrhiza with pine trees (*Pinus* spp.). The relationship is considered to be parasitic with *S. bovinus* and/or pine trees being parasitized by *G. roseus* (Olsson et al., 2000). In this case also the dependent parasite *G. roseus* could be cultured in laboratory when co-cultures with the host bolete was introduced. However, in most cases mycologists fail to mimic nature in the laboratory to the extent that would enable growth of fungi. This is due to our lack of knowledge of the factors that affect fungal growth.

Observing the growth of fungi may be simple, whereas the molecular and physiological processes behind fungal growth are more complex and difficult to observe. These are multi-step processes involving physiological changes at cell level, communication within and between the cells and the biotic and abiotic environment. Fungal growth can be roughly divided into two main stages: (i) germination of the fungal spore and (ii) subsequent filamentous growth that creates the network of hyphae called mycelium (Shaw and Hoch, 2007). It is important to recognize that the growth requirements of an individual species or strain may vary significantly between these two stages, and that growth induction and inhibition represent two sides of the same phenomenon and are therefore treated in parallel below.

### Compounds Influencing Spore Germination in Fungi

The research on growth factors that induce or inhibit fungal spore germination started blooming in the beginning of the twentieth century and focused on plant pathogenic rust fungi attacking crops, ectomycorrhizal (EcM) fungi of forest trees and few selected saprotrophs such as *Agaricus campestris* (Maneval, 1922; Fries, 1966; and references therein). The EcM mushrooms in Agaricomycetes have been particularly challenging to culture and their study forms a great example of the development of growth factor research, both when it comes to spore germination and filamentous growth. Most EcM fungi could not be cultured in axenic cultures without their host plants, trees and shrubs in the first half of the twentieth century which formed a major scientific study question and hindered their application in agriculture and industry. The pioneer in the EcM fungal research was Elias Melin from Uppsala University, Sweden (Lindeberg, 1989). He was especially interested in the growth of mycorrhizal fungi associated with forest trees and created the term M-factor (or factor M; Melin, 1962). With the M-factor Melin (1962) pointed to “growth-promoting metabolites other than B-vitamins and amino acids, which are essential to the growth of tree mycorrhizal fungi.” He applied the term to include both growth promoting effect on mycelial growth and spore germination. Melin’s hunt for the M factor was continued by a fungal physiologist Nils Fries who devoted his research career to studying the growth of filamentous basidiomycetes and spore germination in EcM fungi.

In 1966, Fries reported interesting observations of spore germinations induced by activator organisms. Spores of the gastromycete *Lycoperdon umbrinum* would only germinate when medium filtrate from a preculture of an unknown fungus on synthetic medium was used as a culturing medium (Fries, 1966). He made similar observations using *Rhodotorula mucilaginosa* culture filtrates as an activator of spore germination on spores of *Lycoperdon* and different *Boletus* spp. (currently taxonomically placed in several genera of boletes). Later, it was found out that spores of *Suillus luteus* could also be induced to germinate using an activator organism, such *as Rhodotorula* yeast, that removes a germination inhibiting factor, ammonium ions (Fries, 1976). Germination induction on the other hand was observed when using glutamate or mixture of amino acids in the culturing medium. These observations helped to explain the spore germination stimulation by the activator yeast that, when co-cultured with *Suillus* spores, removes NH4+ from the media and secretes amino acids into the medium. Consequently, the spore germination could be induced in chemically specified and reproducible conditions. This information is extremely useful today when testing media promoting germination of environmental spores in order to detect new biological diversity for NPD. Alternatively, inspired by Fries, potential activator fungi (and other microbes) that are readily cultured from environmental samples could be first cultured and the medium filtrate recycled to be used on the spores from the same environment.

It is unknown to the authors whether the selection of *Rhodotorula* as a co-culturing partner was based on ecological observations of co-occurring taxa of EcM fungi. However, a clearly ecology-based approach to induce the germination of EcM fungal spores practiced at the same time was co-culturing fungi with their hosts or using host extracts to induce the germination. In the tree seedling technique, seedlings are cultured and placed on agar or gelrite medium where spores of tested fungi have been spread (Fries and Swedjemark, 1986). Using this technique, it was shown that basidiospores of *Hebeloma mesophaeum* rapidly germinated in the presence of five different tree species, including trees that are not known to be natural hosts of the fungus (Fries and Swedjemark, 1986). Spores also germinated in the presence of the living tissue of two tree species tested, indicating that unorganized parenchymatic tissue could be used in induction instead of seedlings with roots. Carrot roots and tissue proved to be good inducers that gave equally frequent germination as the tree species tested, whereas several other herbaceous plants failed to induce germination. Additionally, Fries and Swedjemark (1986) showed that the germination-inducing compounds are in the lipophilic fraction of pine root extract, as earlier shown with the same tree species and several other EcM fungi including bolete species and the agaric *Laccaria laccata* (Melin, 1962; Fries, 1985). It is worth noting, however, that the tree seedling technique fails often, and spores of many EcM fungi cannot be brought to germination, which indicates that in addition to compounds leaking from host roots, other growth factors (or inhibitors) possibly from the soil environment are involved in triggering the spore germination.

Co-culturing with bacteria can also be used to induce germination as shown by Ali and Jackson (1989). They isolated bacteria and the fungi from the sporocarps and spores of *Hebeloma crustuliniforme*, *Salix* sp. roots that had *H. crustuliniforme* as mycorrhizal symbiont and birch forest soil with promising results. The spores of this EcM fungus did not germinate on Fries agar medium, but the germination was stimulated by several bacteria and one rust fungus *Tritirachium roseum* associated with the mushroom. It has been also shown with the saprophytic polypore species *Ganoderma applanatum* that spore germination factors can be produced by diverse co-cultured microbes, including bacteria, filamentous basidiomycetes, yeast and filamentous fungi in Ascomycota (Brown and Merrill, 1973). There are few studies on germination-supporting bacterial-fungal interactions (BFIs), but the induction by associated microbes on fungi seems to be common, yet overlooked approach in cultivability and NPD (Ali and Jackson, 1989). One reason for the scarcity of germination studies may be the sparse fungal growth that allows the production of only minute volumes (microliters) of samples to be analyzed. However, even with scanty growth, metabolites responsible for the induction may be extracted, and potentially new metabolites identified with current microanalytical methods utilizing high resolution mass spectrometry instruments.

Other studies have focused on the effect of volatile organic compounds (VOCs), and highlight the versatility of microbial interactions in fungal growth and demonstrates that physical contact or diffusion of growth factors through an agar matrix is not always necessary for successful induction of germination. These studies include the work by McTeague et al. (1959) and Lösel (1964) with basidiomycetes and Oort (1974) who showed that VOCs from the ascomycete *Bretziella fagacearum* (Microascales) triggered the germination of spores of six *Lactarius* species. VOC-triggered growth facilitation can be done using axenic cultures and spores in common gaseous environment on separate growth media. Working with axenic cells is an advantage especially when working with pairs of mycelial cultures that are also shown to interact through VOCs (see following section). Other technologies that are less utilized in the studies on fungal germination factors are the use of chromatography, spectroscopy, and nuclear magnetic resonance (NMR) techniques for the chemical characterization of germination factors. It has been shown using thin-layer chromatography and NMR that abietic acid (as one of many molecules observed in tree seedling root exudates) induced spore germination of four *Suillus* species (Fries, 1987).

Spore germination studies have also been conducted in other groups of plant symbionts. Non-sterile soil paste has given excellent spore germination response in AM fungi and in general their optimal germination conditions correspond to optimal plant growth conditions (Daniels and Trappe, 1980). It has been also shown with individual species, such as *Glomus caledonius*, that spores of AM fungi can germinate on agar medium without their hosts or soil matrix when nutrients and autoclaved plant pieces are present (Hepper, 1979). These results highlight the importance of microbial and plant host interactions for AM fungi spore germination. In the growth of this group of plant-mutualistic fungi, it is not the initiation of spore germination that is most critical step, but rather subsequent branching of hyphae from the germinating spore that has formed a bottleneck for culturing.

Following the initial germination and germ tube formation of an AM spore that can be induced as described above, branching of the hyphae of the germinating spore takes place demonstrating a host recognition response of the fungus (Akiyama and Hayashi, 2006). This process seems to be crucial for the further development of the AM fungus, and the factors that contribute to it have been termed branching factors (BF). Over the last decades several BFs have been identified and characterized, including the strigolactones 5-deoxy-strigol, strigol, sorgolactone, and its synthetic GR7 and GR24 analogs (Akiyama et al., 2005; Besserer et al., 2006). Related and unrelated compounds belonging to other compound classes have been documented also in EcM fungi-host systems where they have been shown to play a similar ecological role in mycorrhizal infection (Lagrange et al., 2001; Herrera-Martínez et al., 2014). The BFs and other mutualism-supporting chemical signals in mycorrhizal relationships form an important study field that is likely to contribute to successful completion of spore germination of symbiotic fungi cultured in host-free axenic cultures.

### Physical Factors Affecting Spore Germination

In parallel with the intensive M-factor investigations of growth supporting chemical compounds, studies on the importance of physical factors affecting spore germination were ongoing. The application of ecologically relevant incubation parameters, such as temperature or pH, were commonly applied to induce fungal growth. This approach is still widely practiced today for NPD. In addition, to the mentioned parameters, large-scale fermentors with control of the gaseous environment have been widely utilized in industrial microbiology, but also small-scale bioreactor systems in laboratory conditions (Duetz, 2007). The two most critical gases in the aerobic fermentation process are oxygen and carbon dioxide.

Oxygen requirements for spore germination vary between species and most likely also between spores from different individuals of a single species. Oxygen requirements are dependent on the incubation or environmental conditions of the spore as well as chemical and physical factors. Different external conditions may be required in different phases of the spore germination, such as swelling or germ-tube production (Goddard, 1935; Wood-Baker, 1955; Sussman and Halvorson, 1966) and internal conditions of the spores (maturity, longevity, dormancy, and vitality) play a role as well (Tabak and Cooke, 1968). Furthermore, spore concentration per volume of medium that may be related to O_2_/CO_2_ requirements or physico-chemical features is important. Gas requirements are connected to the germination vs. spore density phenomenon: higher concentrations of spores inside a medium germinate poorer compared to lower concentrations at the edge of the medium. This seems to be the general rule (Coons, 1916; Weimer, 1917).

Carbon dioxide has been shown to stimulate spore germination in wood-decomposing fungi in moderately elevated concentrations compared to ordinary air (Hintikka, 1970). This is interpreted to be an ecological adaptation to life in or on wood that is releasing CO_2_ when decomposed. CO_2_ has an acidifying effect on culture medium and potential spore germination induction may be due to decreasing pH, rather than CO_2_ itself. However, the wood-decomposing fungi in Basidiomycota showed individual responses to these correlated factors with several species having different responses to CO_2_ at different pH, whereas some fungi had no pH-related difference in germination between pH 3 and 6.5 and under same CO_2_ concentration (Hintikka, 1970). Whereas CO_2_ is a stimulant for spore germination in some fungi, others require high CO_2_ concentration and complete lack of oxygen for the successful formation of viable spores, such as the typical rumen fungi in Neocallimastigomycota (Orpin, 1975; Gruninger et al., 2014).

Allen (1955) showed that floating and soaking under aerobic conditions removes an inhibitor dependent on aerobic metabolism. This inhibitor prevents the germination of uredospores that has an increased effect with spore density. The inhibitor could be deactivated using 2,4-dinitrophenol, methyl napthoquinone, coumarin, and it was demonstrated that this inhibitor is likely trimethylethylene (Forsyth, 1955). Alternative wetting and soaking also induces teliospore germination of rusts (Maneval, 1922; Fries, 1966). Other factors that induce the germination of the teliospores of rust fungi are oxygen supply (no germination without oxygen) and the maturity of spores for which a certain resting period of days-months is required. However, teliospores of some species do not require a resting period (Maneval, 1922).

For the germination of AM fungal spores physical factors such as incubation temperature, pH, and presence of salts have proven important (Mosse, 1959, 1962; Green et al., 1976; Daniels and Trappe, 1980; Tommerup and Kidby, 1980; Hirrel, 1981). These factors need to be within a certain range for a spore of any given species to germinate. Also light has proven critical for the spore germination (Schenck et al., 1975; Watrud et al., 1978). Responses to physical parameters are taxon-specific and ecology-dependent, but in general AM fungi spore germination is reported to take place in incubation temperatures around 20–25C and pH values around 5–8 (Watrud, 1984). AM fungal spores should be incubated for germination both in dark and light, since light is inhibiting the spore germination in some species of AM fungi through unreported mechanisms (Schenck et al., 1975; Watrud et al., 1978).

As seen in more recent literature (Supplementary Table 1) tens of germination factors of fungal spores have been identified since the review of Fries (1966). Progress has been made especially in the study of chemical germination factors of fungi that are relevant for agricultural and forestry applications. These economically important fungi represent a small fraction of the total fungal diversity and the germination factors observed may be highly group-specific and not applicable for fungi with other lifestyles. Progress has also been made in the application of study techniques. In more recent research biochemical separation and spectrometry techniques have been successfully applied to rapidly identify the observed germination factors, although these techniques, coupled with transcriptomics, could be more frequently applied to study the fundamental phenomenon of spore germination. What remains to be done is to widen the selection of fungi studied for spore germination factors. Some saprotrophs are easy to culture (Fries, 1966), while others, e.g., ascomycetes on hard lignicolous substrata grow poorly. Surveys of culture catalogs and molecular data suggests that we have only brought a small fraction of these fungi into cultures (Tedersoo et al., 2014). Obviously, thousands of species of described fungi remain to be cultured from spores from authenticated voucher specimens and screened for novel NPs. Moreover, thousands of species of saprotrophic fungi remain to be cultured and characterized morphologically and molecularly. More germination factors should be identified and used to facilitate germination of spores to initiate laboratory cultures of these fungi that are rich in secondary metabolites and proteins relevant for NP research.

### Compounds and Other Factors Contributing to the Success of Mycelial Growth

The other critical stage in the growth of a fungus is its mycelium. When germinated spores have depleted stored nutrients, and initiate growth, they need to be in the right kind of environment that can sustain the fungus. Extending hyphae absorbing nutrient particles provide the fungus energy that is needed to establish the mycelial network and complete the life cycle of a fungus. Many papers dealing with growth effects involving fungi and other microbes deal with growth inhibition and production of antimicrobial compounds. For example, Sonnenbichler et al. (1994) challenged wood-inhabiting basidiomycetes *Heterobasidion annosum*, *Gloeophyllum sepiarium*, and *Armillaria ostoyae* in co-culture trials with each other, and found out that stimulation of defense mechanisms and growth inhibition was mediated by signal compounds. These were small molecules that the fungi secreted to culture media in low concentrations already in monocultures. When another fungus sensed the presence of the antagonist, it’s synthesis of toxic secondary metabolites was enhanced up to 400-fold highlighting the strong stimulatory response of growth-influencing molecules co-culturing can have in filamentous microbes.

EcM fungi in Basidiomycota are among the best studied group for factors that induce filamentous growth (Supplementary Table 2). Whereas Barnett (1964) summarizes the work done on the growth induction of filamentous mycoparasites belonging to several phyla, Melin (1962) was among the first to show that in addition to co-culturing with living hosts, excised roots had growth-inducing effect on EcM fungi. The growth response toward excised tomato roots, and roots of other distantly related plants (Melin and Rama Das, 1954) was similar, but non-unanimous with some fungal species’ growth not being induced. In line with spore germination studies the unknown factor of filamentous growth from EcM fungi hosts were called Factor M. The M factor was considered to be compounds or a compound consisting of two components, one of which is soluble and the other insoluble in water (Melin, 1954, 1962; Melin and Rama Das, 1954). Later, it has been shown that there is not a single Factor M of filamentous growth, but several growth factors leaking from roots that trigger the growth of EcM fungi. With the help of thin-layer chromatography and nuclear magnetic resonance (NMR) it has been shown that constituents of root exudates including palmitic and stearic acid and cytokinins, such as kinetin, zeatin, and isopentenylaminopurine induce the filamentous growth of many EcM fungi, but also saprotrophic species (Sun and Fries, 1992). This was an important finding, as it gave chemical identities to the Factor M. Moreover, the finding proved that exact concentrations of laboratory-grade single compounds can be used to induce growth instead of amending media with root exudates with significant temporal and geographical variation in concentrations of individual constituents that is likely to lead to reproducibility failures of experiments.

There are relatively few studies utilizing chromatography and spectrometry techniques to reveal the exact chemical identity of the Factor M. Zeng et al. (2003) studied *Brassicaceae* plants and induction of filamentous growth in *Paxillus involutus* and *Pisolithus tinctorius*. Root exudates from eight and two species of *Brassicaceae* plants were shown to induce the growth of *P. involutus* and *P. tinctorius*, respectively. Using high-performance liquid chromatography (HPLC), the authors identified indole glucosinolates in turnip (*Brassica rapa*) roots that were suspected to be responsible for the observed growth induction. However, these suspected growth factors were not isolated and tested as a chemical fraction or pure compounds. The work of Zeng et al. (2003) points to the complexity of interactions in nature where growth factors for EcM fungi may leak into the soil from not only their host plants, but also other surrounding macroorganisms. More studies utilizing modern spectrometry instruments and today’s metabolomics tools would be welcome to chemically identify and characterize growth factors of fungi.

### Growth Factor Studies in Different Groups of Fungi

Melin’s observations on EcM fungi and their growth demands has laid ground for further studies in several groups of fungi. One of the groundbreaking results was the preparation of MMN (modified Melin-Norkrans) medium for culturing of EcM fungi (Marx, 1969). This medium is slightly acidic (pH 5.5) and is composed of complex and simple carbon sources (sucrose, malt extract), salts and minerals [CaCl_2_, NaCl, KH_2_PO_4_, (NH_4_)_2_HPO_4_, MgSO_4_°7H_2_O, FeCL_3_], vitamins (thiamine-HCl, in addition to B vitamins contained in malt extract), and agar and has been widely and successfully applied to the culturing of EcM fungi with modifications (Vuorinen et al., 2015). In addition to carbon and nitrogen sources and necessary minerals required for any microbiological medium, MMN contains the known growth factors glucose (as part of sucrose), thiamine (Supplementary Table 2), and iron. Iron has an important function as catalyst of cellular reactions in fungi and for this reason fungi enhance its uptake from the environment using siderophores that are shown to facilitate growth of a previously uncultured fungus (Johnson, 2008; Beguin, 2010).

One of the plant hosts that has been focused on in EcM studies is the Norway spruce (*Picea abies*). Laiho (1970) studied the growth requirements of *Paxillus involutus*, a facultative EcM symbiont of *P. abies*. He found out that the growth of *P. involutus* is induced by nitrogen in the saprotrophic mode of the fungus. Presence of other microbes (semiaseptic growth) boosted filamentous growth and mycorrhiza formation. In the mycorrhizal modus the fungus was able to form sporocarps and its growth was boosted by thiamine that it needs to acquire heterotrophically. Molina and Palmer (1982) reviewed other EcM fungi with focus on species that are used for inoculation purposes in tree seedling nurseries, such as *Pisolithus tinctorium*, *Cenococcum geophilum*, *Laccaria* sp., and *Thelephora terrestris*. They studied media constituents, macro and micronutrients, mineral compounds, vitamins, as well as physical incubation parameters, and report on components that have contributed to improved filamentous growth. Examples of semisynthetic and synthetic media that have been successfully used to culture EcM are also given. Using some of the media highlighted in Molina and Palmer (1982), Vuorinen et al. (2015) studied the Athelioid clade EcM fungi associated with *P. abies*, including fungi in the genera *Tylopilus*, *Piloderma* and *Amphinema*, and concluded that the best medium to culture them in large-scale is based on silica powder containing 2.5% (w/V) light brewery malt extract supplemented with 0.5-g/l humic acid product and pH adjusted at 5.8. Recent research done to culture beneficial EcM symbionts to support tree seedling growth typically excludes assessing the growth effect of individual factors, single compounds, or mix of compounds. This is logical as literature shows that there is not “one medium to culture them all” and much trying and failing is expected when trying to find optimal mixture of suitable chemical and physiological growth factors. On the other hand, the scarcity of systematic studies on the effect of single growth factors on fungi has hampered the progress in culturing a greater fungal diversity.

It is obvious that induction of filamentous growth (also spore germination) is not functioning according to one mechanism, but there are several factors and multiple mechanisms involved. Some of the mechanisms appear to be genus specific, e.g., the induction of filamentous growth of *Laccaria amethystina* and *L. bicolor* by lipid fraction of pine root exudates (Fries et al., 1985), or germination induction of abietic acid on the spores of four *Suillus* species (Fries et al., 1987). Discovered growth factors have not been tested on a wide selection of fungi belonging to diverse groups in a high-throughput manner, not even within EcM fungi. Each EcM plant host has a mix of compounds in its root exudates. Evidence points toward the existence of multiple growth-inducing factors that selectively induce the growth of multiple species when applied mixed as root exudates, but may have even more taxon specific induction when applied as single compounds (Fries, 1989). Moreover, a single factor may show dual effect on closely related taxa, e.g., abietic acid inducing the filamentous growth of *Suillus granulatus* and inhibiting *S. luteus* and *Laccaria laccata* (Fries, 1989).

Arbuscular mycorrhizal fungi in the subphylum Glomeromycotina (zygomycetes formerly placed in their own phylum Glomeromycota) are another group of plant symbionts that have proven to be recalcitrant toward culturing. Whereas physical and chemical factors inducing spore germination has been detected and successfully applied (see above), subculturing of the mycelium has not been as successful in pure cultures without the host or other symbiotic partners. In a classical work on zygomycete fungi, Ellis (1966) studied the growth requirements of mycoparasitic *Syncephalis* spp. belonging to the lately described Zoopagomycota phylum (Spatafora et al., 2016). Utilizing a co-culturing approach, Ellis reports successful subculturing of the parasite in an axenic culture by inoculating it on pieces of liver that are embedded in a 0.1% tryptone and 0.4% K_2_HPO_4_ agar prepared in tap water and poured over the liver (Ellis, 1966). He determines the pH and temperature tolerance and optimum for five *Syncephalis* species and concludes that the growth factors required for culturing are water-insoluble material of animal liver.

Benny et al. (2016) compiled and summarized the various methods for axenically culturing or co-culturing many of the fungi which were formerly classified as zygomycetes, including Kickxellomycotina, Mortierellomycotina, Mucoromycotina, and Zoopagomycotina. As an example of some of the techniques to increase cultivability of these fungi, species of mycoparasites in the Dimargatales (Kickxellomycotina) can be grown without their hosts by amending media with high protein sources such as egg, beef, and swordfish (Ayers, 1933). The nematode egg pathogen, *Rhopalomyces elegans* can be grown axenically with specialized media containing calf liver and lamb fat (Ellis and Hesseltine, 1962; Ellis, 1963). Other research relevant to cultivability of former zygomycetes, include the key work by Degawa and Tokumasu (1997) who were able to induce zygospore formation in the soil-isolated *Mortierella capitata* by amending media with sterilized arthropods.

More recent successful culturing studies on zygomycetes involve co-culturing with bacteria. The species *Glomus intraradices* and *Rhizophagus irregularis* can be grown in co-culture with the bacterium *Paenibacillus validus* that is facilitating the growth of the fungi (Hildebrandt et al., 2006; Kameoka et al., 2019). Kameoka et al. (2019) studied this growth triggering interaction deeper and showed that (S)-12-methyltetradecanoic acid, a methyl-branched fatty acid that was isolated from the bacterial cultures facilitated hyphae germination from mother spores and led to the formation of secondary spores in the fungal pure culture.

### Volatile Organic Compounds (VOCs) and Growth

Growth factors that diffuse in liquid or solid growth medium have been the focus in studies of growth-related interactions involving fungi. Gaseous components, excluding common gasses such as O_2_, CO_2_ (treated above), have been less studied (e.g., Hiscox and Boddy, 2017). Results on the effect of VOCs on fungal growth concern mainly growth inhibition. These include, e.g., the interaction between the fungal plant pathogen *Verticillium longisporum* and its bacterial antagonist *Paenibacillus polymyxa* (Rybakova et al., 2017). When exposed to each other both produce VOCs with antimicrobial activities. The bacterial antagonist’s VOCs are more effective, including 2-nonanol, 2-Methyl-1-butanol and hexadecadal, and inhibit the growth of the plant pathogen, slow down its metabolism and cause cell death when in direct contact with the pathogen.

Volatile compounds that are chemically related to the compounds mentioned above have also been reported to contribute to the induction of filamentous growth (Suolahti, 1951; Brown and Merrill, 1973). Fries (1961) reports nonanal contributing to induced growth of three species of wood-inhabiting Basidiomycota of the five tested. Applied as liquid solution, heptanal, nonanal, and several related aldehydes had a growth inducing effect on a broader selection of saprophytic and EcM basidiomycetes. Evans et al. (2008) studied intra- and inter-species VOC-mediated growth interactions across four species of wood-saprophytic basidiomycetes. They found out that VOCs from paired cultures of studied species had in most cases a growth effect on *Bjerkandera adusta* cultured above the interacting fungi. Often the interaction was growth suppressing, but in some cases an induction was observed. Of the fungi studied, *Trametes versicolor*, and in individual cases, *B. adusta* and *Hypholoma fasciculare* were growth-induced above paired cultures compared to agar controls and growth above self. The compounds produced in growth supporting interactions included monoterpenes, benzoic acid, alkenols, and a quinolinium-like compound. The effect of these VOCs on the growth of a broader selection of fungi remains to be tested.

### Methodological Considerations for Culture-Based Search of Growth Factors

The factors inducing/inhibiting spore germination and filamentous growth within a single species are not necessarily the same. For example, Fries (1949) showed with several species of *Mycena* that ammonium ion (0.1–0.5% ammonium tartrate) inhibits the spore germination, whereas the presence of ammonium ions in culturing media of mycelium has no inhibitory effect. The spores of *Rhopalomyces elegans* (Zoopagomycota) can be brought to germination using an autoclaved bacterial (*Bacillus cereus*) culture, whereas extensive filamentous growth (and sporulation) can be induced on a medium containing liver and lamb fat (Ellis and Hesseltine, 1962). The fact that different growth factors may be necessary for spore germination and subsequent growth should be taken into consideration when culturing previously uncultured fungal diversity.

Based on earlier studies on fungal spore germination and subsequent growth, it is evident that growth is an interplay between several factors and in many cases one growth factor is not enough for successful growth of a fungus or germination of its spores, if the incubation environment or medium is not otherwise suitable. For example, germination of ascospores of *Byssochlamys fulva* starts at relatively high temperatures and sucrose levels on acid media, but only under very high CO_2_ conditions (Hull, 1939; Tabak and Cooke, 1968). Heat and solvents, e.g., ethanol, furfural, acetic acid, and phenols, have been used to induce spore germination in academic research as well as by pharmaceutical discovery groups to culture ascomycetes for NPD and this has resulted in several new NPs discovered (Warcup and Baker, 1963; Furuya and Naito, 1980; Okuda et al., 2004). The environment also influences growth-inducing microbial interactions that are strain-specific (Hansen and Jakobsen, 1997). Growth may also be concentration-dependent, as in many cases too low or too high concentrations of a growth factor (or spores in germination studies) do not support growth of an individual fungus that requires certain concentration range in the middle of the extremes for growth stimulation (Sun and Fries, 1992). Induction and inhibition can be seen as two sides of the same coin, and in practice the nature of the germination or growth response is dependent on the concentration of the growth factor. Concentration-dependency and combinatory effect of different growth factors on growth induction requires designing large micro-scale experiments in which single media components are different between individual cultures.

When it comes to strategies for finding new fungal NPs, the saprotrophs and opportunistic pathogens, especially in Eurotiales (*Penicillium* and *Aspergillus*), have proven to be excellent sources for new secondary metabolites and consequently been under intensive scrutiny (e.g., Dreyfuss and Chapela, 1994; Jůzlová et al., 1996; Okuda et al., 2004; Keller et al., 2005; Zhai et al., 2016). These creative producers of secondary metabolites have also dominated in the marine NP research in addition to fungi isolated from different parts of mangrove trees (e.g., Nicoletti and Trincone, 2016). Often the isolation and culturing techniques are not well elucidated when new NPs are reported and, consequently, it is hard to evaluate the ecological lifestyle of the cultured fungi (Carroll et al., 2021). This is especially true for symbiotic fungi that possess divergent genetic tools (Miyauchi et al., 2020). We know from earlier studies that ways to culture symbionts can be developed and that many symbionts have high potential to produce new NPs (Dreyfuss and Chapela, 1994; Tkacz, 2000; Okuda et al., 2004; Ahrendt et al., 2018).

Symbiotic fungi are dependent on the metabolism of their host or partner organism. This means they live in a chemical environment that may be harder to mimic in the laboratory than the substratum of a saprotrophic fungus. However, multiple studies report culturing of a host metabolism-dependent fungus using live hosts or host-derived exudates or extracts (e.g., Melin, 1962; Barnett, 1964; Ellis, 1966; Fries and Swedjemark, 1986). Co-culturing with the host often possesses extra challenges (cultivability and viability of the host in the laboratory environment etc.) and adds to the complexity of the experimental setting. Likewise, controlled experiments may be difficult to conduct due to the chemical variation of natural exudates and extracts over time and space. A more far-reaching alternative for the host-dependent culturing would be to isolate and characterize growth factors of different types of symbiotic fungi and test these chemical entities for their ability to induce growth in a broader selection of symbiotic fungi, as nicely demonstrated with the discovery and characterization of mycotrophein that was shown to be a hydroxamate-type siderophore (Barnett and Lilly, 1958; Whaley and Barnett, 1963; Beguin, 2010). Repeating such a workflow in a systematic manner could contribute to characterizing several new growth factors that could be applied to culturing as-yet-uncultured fungi in a reproducible and controlled manner.

The difficulty in applying new growth factors lies in detecting, characterizing, and possibly isolating these in sufficient amounts. In practice, we first need to find out what those specific growth factors are using at least microbiological, chemical, and likely also molecular biology techniques (Hildebrandt et al., 2006; Nichols et al., 2008; D’Onofrio et al., 2010). If we can identify a known compound, it may be possible that it is easily purchased and applied in a suitable concentration to the medium. If the growth factor is unknown or commercially unavailable, it needs to be isolated (or synthesized) in sufficient amounts before applying in growth induction of a hard-to-culture fungus. This simple description of the workflow underlines that the process requires multidisciplinary collaboration and is often not straightforward.

The alternative is to try to predict growth factors (secondary metabolites), such as siderophores used for acquisition of iron from the surroundings (Horowitz et al., 1976; D’Onofrio et al., 2010). Due to the complexity of microbial communities, functions and natural systems in general, a completely random trying and failing approach is comparable to looking for a needle in the haystack, especially if the test for growth factors is not done using high throughput robotics. Random testing of known growth factors should be, when possible, substituted by ecology-driven search with the aim of culturing symbionts (and other as-yet-uncultured fungi). However, knowledge of as-yet-uncultured microscopic symbionts, their ecology and growth requirements is hard to acquire and applying modern omics techniques to the study system may become a necessity.

## Metabolic Basis of Cultivability Based on Results Obtained Using Sequence-Based and Omics Methods

Genomics, metagenomics and single-cell genomics techniques are widely used in genome mining to identify talented producers of NPs (Ahrendt et al., 2018; Blin et al., 2019; Youngblut et al., 2020), but few researchers have applied these techniques to increase the pool of microbes that can be cultured. The use of omics-based methods is providing a new set of tools in the search for culturing previously uncultivated microbes including fungi for NPD (Omsland et al., 2013; Ahrendt et al., 2018). Genes, whole metabolic pathways, and transcripts or lack thereof can be used to discern the metabolic capabilities of a particular fungus, which can then in turn inform the production of new or amended media conditions for cultivation. A great advantage of the tools presented is that they can also be applied culture-independently for extracting data of microbial growth from single cells to multispecies communities. However, natural communities are often highly complex and omics data from such communities is too challenging for untargeted analyses of features that induce growth of as-yet-uncultured fungi. An isolation or culture enrichment step is needed to reduce the complexity of the system and restrict the culturing to a selection of community members (one to several) that are likely to include so called helper species that facilitate the growth of associated microbes. The consortium *in vitro* can then be sampled for one or preferably several types of omics data and analyzed for growth supporting or inhibiting features.

The first study to take advantage of omics derived metabolic requirements to culture a member of a previously uncultured lineage of bacteria, used a fraction of a metagenome sequence to identify a gene involved in nitrogen fixation (Tyson et al., 2005). This discovery provided enough information for those researchers to use a targeted, nitrogen-free medium to culture and isolate that bacterium. Likewise, using data gleaned from traditional approaches and reconstructed metabolic pathways based on whole genome data, researchers were able to axenically culture the obligate intracellular pathogen, *Coxiella burnetii*, which causes Q fever in humans (Omsland et al., 2013). A similar metagenome information assisted culturing of previously uncultured fungi remains to be published, but in data generation, analysis tools and computational resources there is nothing in the way to conduct this type of study already today making this a relevant future strategy in fungal NPD.

Obtaining a genome for species from a complex sample in which there are multiple taxa present can be challenging. It is possible, however, to use *in silico* techniques to separate target genomes from a metagenome containing genetic material from multiple organisms (Dick et al., 2009; Dodsworth et al., 2013; Chen L.X. et al., 2020). In fungal taxa, at least one genome from metagenomic data from an obligate intracellular pathogen, *Paramicrosporidium saccamoebae* (Cryptomycota), has been produced, resulting in a nearly whole genome sequence (Quandt et al., 2017), and several metabolic deficiencies were identified. For example, *P. saccamoebae* lacks genes involved in amino acid and nucleotide biosynthesis, presumably because it acquires these essential building blocks from its host. Providing pathogens such as *P. saccamoebae* with essential amino acids and nucleotides could potentially improve efforts to cultivate them, although their extreme amount of gene losses may be too difficult to overcome. Genomes have also been generated from fungal fruiting bodies of difficult to culture fungi (e.g., Quandt et al., 2015; Nguyen et al., 2017; Chang et al., 2019). None of these later studies focused on using these metagenome derived genomes to examine their metabolic deficiencies which may prevent them from readily growing in axenic culture, but perhaps by examining common deficiencies in certain lineages or ecological niches, researchers could benefit from collation of this metagenome-derived metabolic data to improve their cultivability.

Single cell genomics provides a different approach for extracting genomes from environmental samples (Woyke et al., 2017). Recently, the first study to employ such methods in fungi, identified commonalities in missing metabolic pathways among unculturable fungi, including pathways involved in assimilatory sulfate reduction and the production of biotin, and thiamine (Ahrendt et al., 2018), that are known to be growth factors in many fungi (Supplementary Table 2). This finding is supportive of traditional media amendment approaches (including the addition of ox bile which is rich in these compounds; see above) for some of the early diverging fungi examined in that study (Ellis, 1966). Although Ahrendt et al. (2018) were unable to grow some of their taxa in media made with these amendments, at least one species, *Dimargaris cristalligena*, grew faster and more abundantly on the amended media, although it was unable to sporulate. That study also successfully used the single cell genomics approach to identify a massive expansion of secondary metabolite gene clusters in the aforementioned mycoparasite, *D. cristalligena*, demonstrating that symbiotic fungi may be prolific producers of novel NPs. Although single cell studies are becoming more frequent in the mycological literature (e.g., Davis et al., 2019a, b; Chen M. et al., 2020; Ciobanu et al., 2021), no others have utilized the data for cultivating as-yet unculturable fungi. Even if culturing is possible under the most ideal conditions, it is worth noting that some fungi may have an intrinsically slow growth rate, and this needs to be taken into consideration as always when deciding incubation times and when considering hurdles to isolating novel NPs from such slow-growing species.

Another feasible method for utilizing high-throughput sequencing data in the quest for culturing environmental fungi is metatranscriptomics. By sequencing all mRNA transcripts from a particular environment, information about the required nutrient conditions or growth triggering factors can be discerned. This approach has already been successfully employed in culturing an obligate symbiotic bacterium found in the gut of a leech (Bomar et al., 2011). By analyzing the metatranscriptome of the leech gut, those authors found specific nutrient requirements of the most dominant bacterium and amended media which enabled its isolation. To date, there are no published accounts of using metatranscriptomics in this way in fungi.

Metabolomics is one tool in the omics toolkit that can be applied for extracting information of natural communities. Due to the complexity of natural communities the target of metabolomics analyses is usually a pair or a small consortium of species. One of the most promising methods to extract information on growth supporting chemical interactions is single-cell metabolomics. Extracting chemical data on cellular metabolome would provide detailed information on the growth supporting microbial interactions. As reviewed elsewhere, this field is in its infancy and under rapid development (Duncan et al., 2019). There are several existing techniques for doing single-cell metabolomics with severe limitations that hinder drawing biologically relevant conclusions. The limitations are related to low detection limits, distinguishing between technical and biological variability of samples, higher sensitivity to detect low abundant metabolites and the quality of software and reference databases (Duncan et al., 2019).

For culture-dependent approaches a particularly useful non-omics technique is mass spectrometry imaging (MSI) of cultures grown on solid medium. In MSI, the two-dimensional spatial distribution of chemical compounds in a sample can be determined, without the need for staining or labeling. The technique has been widely used in biomedical studies and biomarker discovery due to its distinct ability to analyze living systems and to visualize the analysis results with the retained spatial information of chemical entities (Arentz et al., 2017). Recently, MSI has been used in various studies of microbial communities including inter-species metabolic exchange, chemical output of marine microbial assemblages and cross-kingdom interactions (Yang et al., 2011; Moree et al., 2012). Despite its large potential in studying chemical interactions, MSI has not been systematically utilized in cultivability studies.

The use of any omics-based data for improving cultivability of fungi does not require the initial authors or originators of these data to be the ones to take advantage of them. Once published and publicly available, any researchers interested in mining these genomes, metagenomes, metatranscriptomes, or metabolomes for clues into the metabolic deficiencies in species of interest can do so, with proper citation to the original authors. More collaborations between researchers with expertise in comparative genomics and those interested in culturing fungi could greatly move the field forward.

## Ways of Increasing Cultivability Using Top-Down and Bottom-Up Approaches

**FIGURE 2:**
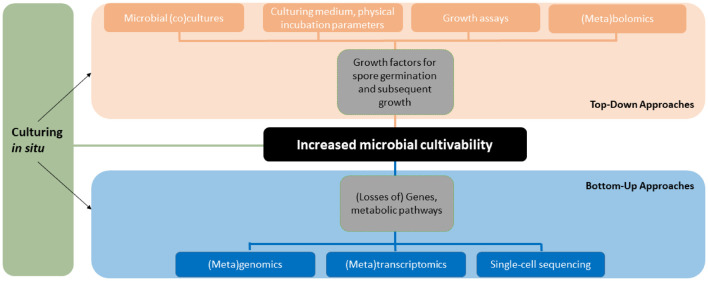
Overview of the methodology and approaches to study fungal cultivability. The figure shows how different techniques relate to the approaches and clarifies the terminology used in this paper. The tools and approaches can be used in different order, combinations and in parallel.

We have provided more than one hundred known growth factors of fungi and exemplified techniques how recalcitrantly cultured or as-yet-uncultured fungi can be cultured based on published literature. The list of mentioned growth factors and techniques is far from exhaustive and numerous new chemical compounds and culturing techniques are likely to be discovered in the future. The approaches of studying and finding factors that increase fungal cultivability can be classified into top-down and bottom-up categories similarly to prevailing strategies in NPD (Figure 2).

As demonstrated by the Swedish pioneers of fungal cultivability, Melin and Fries, simple techniques including co-culturing and ecological media selection utilizing metabolites available in the host or growth substrates combined with chromatography and spectroscopy methods can be used to tease out growth factors that either boost spore germination, subsequent growth or both. These top-down approaches take chemical compounds as a starting point in the search for new growth factors. The secondary metabolites, peptides or other compounds can be extracted from macrohosts or cultures of helper microbes in mixtures or isolating single compounds. The compounds can be tested for growth inducing activity in assays with target cells one wants to culture (Nichols et al., 2008). In untargeted work with no before-hand knowledge of the active compound(s), the growth inducing activity can be followed through cycles of activity testing and fractionation of complex extracts into less complex fractions from which the compounds can be identified using mass spectrometry and possibly isolated in sufficient amounts. The described assay guided isolation of growth factors is similar to bioassay guided isolation of NPs that is a standard procedure in NP research.

A bottom-up approach takes genes, BGCs or metabolic pathways as a starting point and with the use of omics tools allows the user to gain insight into the metabolism and biosynthetic capability of a fungus. This information can be used to design culturing media that complement for the lack of gene or metabolism products, i.e., growth factors, the fungus cannot synthesize itself. Combined with high-throughput culturing and imaging analyses the bottom-up approach could be utilized to effectively reveal new growth factors in a wide diversity of genome sequenced fungi associated with different hosts or substrates.

### Developments in Laboratory Culturing

Over the past several decades, a few laboratory-based methods have been successfully used to culture rare or slow growing fungi and bacteria. For soil fungi, particle filtration or soil washing methods have enabled the isolation of many fungi (Parkinson and Williams, 1960; Gams and Domsch, 1967). However, this type of technique was time consuming, limiting the number of samples that could be analyzed. Dilution to extinction is a similar technique that involves serial dilutions of samples to reduce the density of different microbial inocula, and can be combined with the use of microwell culturing to increase the throughput compared to particle filtration. First explored in bacteria (Connon and Giovannoni, 2002; Bruns et al., 2003), dilution to extinction has led to the isolation of hundreds of fungi, some of which have represented novel diversity (Collado et al., 2007; Unterseher and Schnittler, 2009). These methods have continued to be expanded upon in recent years and the new technologies, discussed below, are providing new opportunities to cultivate even more fungi and mine them for natural products.

Microfluidics-based culturing techniques are one such method that enable controlled culturing of single cells or microbial consortia. These techniques have been used to study, e.g., biofilm formation, degradation of polycyclic aromatic hydrocarbons and other microbiological processes (Unterseher and Schnittler, 2009; Jiang et al., 2016; Pérez-Rodríguez et al., 2021), but also bacterial cultivability (Zengler et al., 2002, 2005; Park et al., 2011). Microfluidics have a huge application potential to study microbial cultivability as reviewed elsewhere (Stewart, 2012; Nai and Meyer, 2018; Lewis et al., 2020; and references therein). Advantages for growth factor studies include controlled handling of liquid culturing medium including sampling for both top-down and bottom-up analyses. Microfluidics systems with circulating nutrition medium also allow controlled and repeated spiking of the medium with potential growth factors. Often the systems are connected to computer software and microscope allowing real-time monitoring and visualization of growth of individual species and the whole consortium. The high cell density per unit volume, especially in cells encapsulated in microdroplets, may inherently induce the growth of microbes that can be considered as a limitation of these techniques for identifying new growth factors (Boedicker et al., 2009; Stewart, 2012).

Controlled hyphal growth of different species of fungi in microfluidics systems poses a methodological challenge, if cell movement within the microfluidics device is not prevented with robust means and is likely to cause problems in successful execution of experiments. The problems are related to the apical growth and foraging behavior of hyphae and tendency for asexual sporulation in culture combined with varying growth rates. Aleklett et al. (2021) shows with seven species of Agaricales mushrooms species-specific growth responses of fungal hyphae when facing mechanical obstacles in a microfluidics device the authors termed Obstacle Chip. Fungal hyphae show great plasticity and navigation in narrow channels and complex structures are solved differently in various species. Some fungi grow faster in narrow channels, whereas others may slow down the growth rate. When facing obstacles, fungi may alter growth morphology to thin non-branching foraging hyphae with short lateral branches, whereas other species may increase branching and produce a denser network of hyphae. Hyphae may also apply tip force, causing pressure against materials and break out from the culturing channel. Several of the tested species also formed spores toward the end of the experiment that may easily be transported in the flow of a microfluidics device and thus impose increased risk of cross-contamination. The incompletely understood foraging behavior of fungal hyphae makes culturing multiple species at microscopic vicinity to each other risky, if cross-contaminations are to be avoided. It is also likely that we are still unaware of some challenges of culturing filamentous fungi in microfluidics, since this study direction is new (Soufan et al., 2018; Held et al., 2019).

Culturing of filamentous fungi in microfluidics devices possesses challenges that may be solved using technological innovations such as incorporating semipermeable membranes in the devices and making them robust enough to withstand hyphal tip pressure. Cross-contaminations may impair our ability to identify the member in the microbial consortium that is producing a chemical growth factor or expressing a gene transcript that is overexpressed in conditions when growth is triggered. This may force researchers to focus more to the consortium as a cultured entity and comparing different consortia.

Another modification of the dilution to extinction method combined with micro-culturing which offers an alternative to microfluidics is the use of miniaturized culture chips that allows the growth of up to hundreds of thousands of colonies per cm^2^ (Ingham et al., 2007; Ge et al., 2016; Liang et al., 2020). One such instrument is the GALT Prospector, a high-throughput technique for microbial isolation from human or environmental samples (Liang et al., 2020). Essentially, dilutions of environmental samples are loaded onto nanoscale microwells, such that a single well contains only, on average, one cell. Wells with growing cells are identified using fluorescent imaging, and then targeted and transferred to standard 96 or 384 plates for downstream analysis. There is no reason that such technology could not be used to isolate environmental fungi, especially those that are small and less abundant or are frequently outgrown by faster growing fungi in traditional plating techniques. It remains to be tested how effective this instrument can be at culturing fungi. Especially fungal cell density and cell size are obvious challenges in using this type of technique. The cell numbers of fungi in environmental samples are approximately thousands time less than that of bacteria and cell sizes on average 10×–100× larger (Gaertner, 1968, 1969; Maranger and Bird, 1995). Consequently, fungal cells should be effectively separated from bacterial cells before inoculating the microwells, or alternatively antibiotics used in the media to suppress bacterial growth. While larger cells and fragments of many fungal species will be too large to be cultured via this technique, many other species of fungi do have propagules or fragments that are in the 1–10 μm range which would be ideal targets for these methods developed in bacterial systems.

An older microbial colony handling platform that has been successfully applied in bacterial culturomics and bacterial cultivability research is the QPix platform (Geesink et al., 2018). The advantage of this platform is that it can pick and inoculate microbial colonies between different formats of solid and liquid cultures. The plating function is also available in other automated platforms (King et al., 2006). Compared to the GALT prospector the first mentioned platform is more versatile and the diffusion of growth factors can be allowed through solid agar-based medium in Petri dish or higher volume culture trays. Early sporulating isolates cause problems that need to be dealt with by early handling of the colonies, weeding of early emerging colonies with a scapel or soldering gum (Dreyfuss, 1986; Dreyfuss and Chapela, 1994), suppressing the sporulation using chemicals, such as diaminobutanone (Guzmán-de-Peña et al., 1998) or focus the work on non-sporulating fungal isolates. Inoculating fungi from liquid cultures may appear problematic due to mycelium clogging the pipette tips and causing spills in inoculation operations. However, this can be avoided by adding glass obstacles to agitated cultures that shear the mycelium resulting in a homogenous mycelial suspension (Duetz et al., 2010).

### *In situ* Culturing

Besides the *in vitro* and *in silico* techniques described above, culturing devices or aides can be brought into the natural environment to grow fungi before taking the devices with growing cell numbers into the laboratory. This has been successfully done by developing *in situ* culturing and baiting techniques that take advantage of uncharacterized natural growth factors by allowing them to freely diffuse to individual cells grown in compartments of the device through semipermeable membranes. When first cultured *in situ*, isolated microbes can be brought into laboratory and domesticated (Kaeberlein et al., 2002; Bollmann et al., 2007; Nichols et al., 2008). In laboratory, the growth factors can be studied in a controlled and simplified system using the bottom-up or top-down approaches mentioned above. Most of the culturing techniques have been applied to bacteria only and we have little published literature documenting how well the techniques work with filamentous fungi. Here, a short summary of developed methods is included focusing on devices that have not been included in recent reviews (Nai and Meyer, 2018; Lewis et al., 2020).

The *in situ* culturing device that has received most attention is the Ichip that was used to isolate a promising antibiotic (Nichols et al., 2010; Ling et al., 2015). This device is basically a high throughput version of a diffusion chamber that is based on incubating cells in solid medium sandwiched between semipermeable membranes that allow growth factors to diffuse to cells. Originally introduced by Kaeberlein et al. (2002) this principle has been applied to culture previously uncultured or recalcitrantly cultured bacteria and obtain new diversity (Bollmann et al., 2007). For optimal filamentous growth of fungi that may be restricted by the small space of growth and tendency of fungi to stick to the walls of the culturing devices (cf. Duetz et al., 2010), the *in situ* devices may be optimized by reducing the number and increasing the size of the growth compartments. There are multiple manufactured modifications of the diffusion chamber principle and you can even build these devices yourself (Epstein et al., 2010). The original construction of the microbial chamber has been modified using different materials and 3D printing (Wilson et al., 2019). Other diffusion-based techniques build upon porous and semi-permeable membranes as well, such as the soil substrate membrane system that Svenning et al. (2003) used to culture methanotrophs or the hollow-fiber membrane chamber (Aoi et al., 2009). The culture chamber introduced by Thøgersen et al. (2018) is also a diffusion-based device where growth factors from a helper microbe or a particular (mix of) chemical compound(s) inside the chamber diffuse through a porous membrane allowing enrichment of microbes on the outside.

The Ichip device allows for massive parallel culturing in microscale microbial chambers. Higher throughput is a desired feature in the hunt for new cultured diversity and consequently there are further modifications of the Ichip (Marcolefas et al., 2019). Further miniaturization of the Ichip increases the throughput even more and allows for culturing in even smaller volumes (Epstein et al., 2010). The advantages of the miniaturization include increased diffusion and higher throughput, but its major drawback with fungi may be that the tip force of fungal hyphae may cause leaks in thin layers of materials and membranes leading to cross-contaminations (Aleklett et al., 2021).

Baiting is another long-used, *in situ* method for trapping fungi in their natural habitat and isolating them into culture. Baits are made from a variety of substrata (e.g., seedlings, plant tissues, wood, sand, animal tissues, live animals) and are typically used to target pathogens of plants (Pfenning and Abreu, 2006) or animals (Drechsler, 1941; Ajello, 1956) but are also frequently used to bait saprobic chytrid fungi (Karling, 1945, 1946), marine ascomycetes (Kohlmeyer and Kohlmeyer, 1979; Overy et al., 2014), and mycorrhizal fungi (Brundrett et al., 2003). One of the advantages of baiting using a specific substratum is that something about the ecology or nutritional resources can be inferred, whereas “blind” culturing using dilution to extinction methods on a generic medium (e.g., PDA) conveys almost nothing about what that organism is capable of doing in the environment. Hence, sampling strategies that target specialized ecologies can help to maximize success in NPD.

When using the traditional diffusion chambers or any of their high-throughput versions, microbial cells are preinoculated into the culturing matrix in the laboratory and the cells encased in the device are brought to nature for culturing. The principle of diffusion chambers can also be used to trap microbes one wants to isolate. In microbial traps, diffusion chambers with cell-free growth medium sandwiched between semipermeable membranes is placed into the field and microbes are allowed to grow into the device by using a bigger pore-size membrane facing the substratum from which the microbes are isolated from. This technique is especially suitable for trapping filamentous microbes such as fungi and Actinobacteria from soft substrata: the traps can be seamlessly embedded into substrata, e.g., sediments or soils (Gavrish et al., 2008; Epstein et al., 2010). Filamentous microbes including fungi and Actinobacteria are superior producers of novel secondary metabolites and for this reason microbial traps seem especially appropriate in microbial NPD.

## Discussion

Fungal cultivability as an ecophysiological phenomenon has been little studied during the last decades. Whereas fungal cultivation studies have increased in numbers even after the discovery and development of different omics techniques, the cultivability as a phenomenon has received little attention when compared to the end half of the twentieth century. Several research gaps that are relevant for NPD can be identified and these are discussed below.

Filamentous fungi are excellent producers of bioactive NPs. Still, most of the cultivability research aiming to increase the diversity for NPD has been done on bacteria. There are likely to be many historical and methodological reasons for this, such as the higher numbers of microbiologists working with bacteria compared to fungi or the higher taxon richness or smaller genomes of bacteria that makes bacteria more tractable objectives. Fungal ecologists and taxonomists should be enlisted more often in NPD to fill the knowledge and technical gaps and lead the way to more successes based on fungi-derived NPs.

Fungi and Bacteria have coexisted and coevolved for hundreds of millions of years and during this time have developed complex interactions. Considering the high taxon richness and diversity in both groups, it can be said that these interactions remain sorely underutilized in NPD. NPD has considered the relationship of fungi with their growth substrates or macrohosts when enhancing the growth of fungi in laboratory, but microbial cross-kingdom interactions remain less explored and utilized. Bacterial germination induction of EcM fungal spores seems to be common (Ali and Jackson, 1989), but the knowledge remains underutilized to grow EcM or other recalcitrantly cultured groups of fungi for NPD. It has been shown that fungi colonizing dead wood are one of the drivers of the composition of bacterial community (Johnston et al., 2019). Especially, bacteria in the Burkholderiaceae family increase in abundance in wood colonized by certain basidiomycetes compared to controls without fungi. In laboratory trials, the inclusion of bacteria belonging to Burkholderiaceae is shown to affect the growth of basidiomycetous fungi and affecting competitive performance of paired species in fungal-fungal interactions (Christofides et al., 2020). Although we have just scratched the surface of cross-kingdom growth affecting interactions in community dynamics, there certainly are many documented cases of growth promoting cross-kingdom interactions between two species (Frey-Klett et al., 2011; Scherlach et al., 2013; Scherlach and Hertweck, 2020). We encourage researchers in NPD to design studies that screen these delicate interactions for novel chemistry in parallel with harvesting the chemical diversity of ubiquitous saprotrophs and parasites of macroeukaryotes.

The tools to study microbial interactions are better than ever and should be combined with the available taxonomical and ecological knowledge. The integrated omics enabled approach for NPD combining top-down and bottom-up approaches can be utilized in many ways. For example, researchers can use metagenomics and/or transcriptomics to detect dominant, cultivable fungi in a given environment that are easily isolated and cultured. These helper fungi and the metabolism products synthesized during growth can be used to induce the growth of environmental cells from the same environment in laboratory in ways suggested by Fries (1987) or applied by Nichols et al. (2008) with bacteria. Alternatively, one can use microbial traps with helper organisms that are brought to the natural habitat to bait for associated filamentous microbes. That can be domesticated to culture using several rounds of co-culturing or media design based on data from omics approaches.

One of the obvious knowledge gaps is lack of a databases that contains information about fungi and media that can support their growth. The list of growth factors we provided can be used to enhance fungal growth in NPD. Doing this in a high throughput manner utilizing robotics may provide good results, but would remain an empirical search that can be compared to looking for a needle in a haystack due to the countless interactions and chemical products involved. What is needed to bridge the gap is a systematic study on media-fungus pairings that is made available to the NPD community in the form of a searchable database. Similar to the KOMODO database (Oberhardt et al., 2015) such a work could reveal more taxon-specific growth factors and allow testing the hypotheses of whether ecology and evolutionary relationships can be used to predict growth in fungi and whether a phylogenetic marker, such as the 16S in Bacteria, could be used as an indicator of growth supporting culturing medium of as-yet-uncultured fungi.

High-throughput culturing devices such as the Ichip could be easily turned into microbial traps by not inoculating the cells into the device and by applying a bigger-pored membrane to the side of the device facing the substrate the microbes are isolated from. Obviously also other techniques taking advantage of the apical growth of fungi could be developed. Optimally, the *in situ* culturing workflows should be designed to facilitate culturomics studies and automatic handling of microbial colonies using robotics.

The challenge of separating fungal cells from bacterial cells in environmental samples and targeting the cultivation efforts to fungi can be solved using single cell sorting and handling in addition to the use of antibiotics to curb bacterial growth (Huys and Raes, 2018; Lewis et al., 2020). There are multiple tools available that can be used in combinations including fluorescence *in situ* hybridization (FISH) to label cells of interest with fluorescence probes, fluorescence-activated cell sorting (FACS), optical tweezers, or even low-tech option such as dental files (Davis et al., 2019a) for picking out the cells of interest from environmental sample for downstream sampling and analyses. In the applied FISH protocols, cells are fixed and lose their viability which makes them unsuitable for further culturing. A recent live-FISH approach overcomes this limitation and provides a promising tool for fungal cultivability studies and NPD (Batani et al., 2019). Another option for taxonomic selection of viable single cells is reverse genomics that can be used for reconstructing single genomes from environmental metagenomes. The complete or nearly complete metagenome assembled genomes (MAGs) can be analyzed *in silico* and their extracellular proteins predicted. These predictions can be used to design fluorescently tagged antibodies that then will bind target cells in complex environmental cell mixtures. Cross et al. (2019) used the technique described above with FACS to culture a previously uncultured bacterium from a single-cell. Alternatively, Raman-activated cells sorting can be used for isolation of viable cells (Lau et al., 2008). Some of the tools mentioned above are also applied to fungal cells and their integration in isolation and culturing workflows or omics analysis has resulted in important insights into fungal ecology and biodiversity (e.g., Jones et al., 2011).

Single-cell omics offers great promise to study fungal cultivability with the aim of isolating new diversity to culture and use cultures for NPD. While single-cell omics are applied to individual cells, the metaomics tools can be used to analyze the metabolic or transcriptional foundation of growth or lack of it in co-culture or not-too-complex microbial consortia. There also remains to be a wealth of NPs to be discovered simply using a combination of high throughput cell sorting, dilution to extinction methods combined with genomics (Ondeyka et al., 2009; Xu et al., 2011; Ortíz-López et al., 2015). Application of these omics tools to fungi remain restricted due to the bigger size and complexity of eukaryotic genomes including repetitive elements and exon-intron structure. However, the omics toolkit of fungi and the data storage and analysis infrastructure is rapidly expanding lowering the threshold to apply these tools to fungi (Grigoriev et al., 2011). Already today metagenomics has shed light into soil habitats with highest potential for antibiotics production (Bahram et al., 2010) and single-cell sequenced genomes or MAGs analyzed for biosynthetic potential to focus culturing efforts to most prolific or potential producers of desired NPs (Ahrendt et al., 2018; Blin et al., 2019).

## Conclusion

Increased fungal cultivability is crucial for future innovations. A multitude of fungal species remain uncultured, and this unutilized diversity contains a vast pool of chemical entities relevant for microbiology, NPD and related fundamental and applied fields of science. Culturing even a slightly larger fraction of the as-yet-uncultured fungi would be a giant leap toward new microbe-based innovations. These advances will require uses of multiple culturing techniques developed since early twentieth century and combining these with new culturing approaches, state-of-the-art analytical chemistry and omics tools. This integrative approach requires an interacting multidisciplinary research community that embraces interacting microbial communities including microbes from all domains of life.

## Author Contributions

TR conceived the idea for the manuscript and compiled the tables on growth factors. TR and CAQ wrote the manuscript and approved its final version. Both authors contributed to the article and approved the submitted version.

## Conflict of Interest

The authors declare that the research was conducted in the absence of any commercial or financial relationships that could be construed as a potential conflict of interest.

## Publisher’s Note

All claims expressed in this article are solely those of the authors and do not necessarily represent those of their affiliated organizations, or those of the publisher, the editors and the reviewers. Any product that may be evaluated in this article, or claim that may be made by its manufacturer, is not guaranteed or endorsed by the publisher.

## Abbreviations

AM: arbuscular mycorrhizal
BGC: biosynthetic gene cluster
EcM: ectomycorrhizal
NP: natural product
NPD: natural products discovery
OSMAC: One-strain-many-compounds approach.
