# Supplementary material for: Improving Fungal Cultivability for Natural Products Discovery
Source: Front Microbiol. 2021 Sep 16;12:706044. doi: 10.3389/fmicb.2021.706044 (PMC8481835; doi:10.3389/fmicb.2021.706044)
Supplement: Supplementary file 1 [file Data_Sheet_1.docx]

**Supplementary References**

These references appear in the Supplementary Tables S1 and S2 listing the growth factors of fungi.

Induction of spore germination (Supplementary Table S1):

(Duggar, 1901; Ferguson, 1902; Welsford, 1907; Falck, 1912; Thiel and Weiss, 1920; Brown, 1922; Maneval, 1922; Noble, 1924; Uppal, 1924; Schaffnit, 1926; Uppal, 1926; Rippel and Bortels, 1927; Leszczenko, 1928; Platz, 1928; Sibilia, 1930; Rabinovitz-Sereni, 1931; Wilhelm, 1931; Stakman et al., 1934; Goddard, 1935; Ling, 1940; Fries, 1942; Schopfer, 1942; Kehl, 1943; Emerson, 1948; Fries, 1949; von Guttenberg and Strutz, 1952; Machlis and Ossia, 1953; Sussman, 1953; Bretzloff Jr, 1954; Bukowski, 1954; Emerson, 1954; Yu, 1954; Allen, 1955; Gassner and Niemann, 1955; Turel, 1955; Tylutki, 1955; Wood-Baker, 1955; Niemann, 1956; French and Weintraub, 1957; Niemann, 1957; Sumere et al., 1957; Yanagita, 1957; Farkas and Ledingham, 1959; McTeague et al., 1959; Mosse, 1959; French, 1961; Ellis and Hesseltine, 1962; Melin, 1962; Lösel, 1964; Watson, 1964; Buston et al., 1966; Fries, 1966; Yates et al., 1968; Fletcher and Morton, 1970; Hintikka, 1970; Marx and Ross, 1970; Brown and Merrill, 1973; Oort, 1974; Stack et al., 1975; Fries, 1976; Horowitz et al., 1976; Fries, 1978; Fries and Birraux, 1980; Birraux and Fries, 1981; McCracken, 1982; Bjurman and Fries, 1984; Azcon-Aguilar et al., 1986; Fries and Swedjemark, 1986; Mayo et al., 1986; Fries et al., 1987; Krishna and Sharma, 1987; Ali and Jackson, 1988; 1989; Fries, 1989; Ruan et al., 1995; Akiyama et al., 2005; Besserer et al., 2006; Kikuchi et al., 2007; Page et al., 2017)

Induction of filamentous growth (Supplementary Table S2):

(Ayers, 1933; Kögl and Fries, 1937; Haskins and Weston Jr, 1950; Melin and Rama Das, 1954; Barnett and Lilly, 1958; Melin, 1962; Whaley and Barnett, 1963; Wardle and Schisler, 1969; Laiho, 1970; Dijkstra et al., 1972; Lehrian et al., 1976; Schisler and Volkoff, 1977; Bardet, 1982; Fries, 1985; 1989; Duponnois and Garbaye, 1990; Sun and Fries, 1992; Tillotson et al., 1998; Currie et al., 1999; Rikhvanov et al., 1999; Lagrange et al., 2001; Hildebrandt et al., 2002; Zeng et al., 2003; Maier et al., 2004; Heilmann-Clausen and Boddy, 2005; Hildebrandt et al., 2006; Riedlinger et al., 2006; Schrey et al., 2007; Xu et al., 2008; Adams et al., 2009; Beguin, 2010; Deveau et al., 2010; Herrera-Martínez et al., 2014; Kameoka et al., 2019; Sbaraini et al., 2021)

Adams, A., Currie, C., Cardoza, Y., Klepzig, K., and Raffa, K. (2009). Effects of symbiotic bacteria and tree chemistry on the growth and reproduction of bark beetle fungal symbionts. *Canadian Journal of Forest Research* 39(6)**,** 1133-1147.

Akiyama, K., Matsuzaki, K.-i., and Hayashi, H. (2005). Plant sesquiterpenes induce hyphal branching in arbuscular mycorrhizal fungi. *Nature* 435(7043)**,** 824-827.

Ali, N.A., and Jackson, R.M. (1988). Effects of plant roots and their exudates on germination of spores of ectomycorrhizal fungi. *Transactions of the British Mycological Society* 91(2)**,** 253-260.

Ali, N.A., and Jackson, R.M. (1989). Stimulation of germination of spores of some ectomycorrhizal fungi by other micro-organisms. *Mycological Research* 93(2)**,** 182-186.

Allen, P.J. (1955). The role of a self-inhibitor in the germination of rust uredospores. *Phytopathology* 45(5)**,** 259-266.

Ayers, T.T. (1933). Growth of Dispira cornuta in artificial culture. *Mycologia* 25(5)**,** 333-341.

Azcon-Aguilar, C., Diaz-Rodriguez, R.M., and Barea, J.-M. (1986). Effect of soil micro-organisms on spore germination and growth of the vesicular-arbuscular mycorrhizal fungus Glomus mosseae. *Transactions of the British Mycological Society* 86(2)**,** 337-340.

Bardet, M. (1982). *MSc thesis.* Uppsala University.

Barnett, H., and Lilly, V. (1958). Parasitism of Calcarisporium parasiticum on species of Physalospora and related fungi. *West Virginia Agricultural and Forestry Experiment Station Bulletins* 420T.

Beguin, H. (2010). Tritirachium egenum, a thiamine-and siderophore-auxotrophic fungal species isolated from a Penicillium rugulosum. *FEMS microbiology ecology* 74(1)**,** 165-173.

Besserer, A., Puech-Pagès, V., Kiefer, P., Gomez-Roldan, V., Jauneau, A., Roy, S., et al. (2006). Strigolactones stimulate arbuscular mycorrhizal fungi by activating mitochondria. *PLoS Biol* 4(7)**,** e226.

Birraux, D., and Fries, N. (1981). Germination of Thelephora terrestris basidiospores. *Canadian Journal of Botany* 59(11)**,** 2062-2064.

Bjurman, J., and Fries, N. (1984). Purification and properties of the germination‐inducing factor in the ectomycorrhizal fungus Leccinum aurantiacum (Boletaceae). *Physiologia plantarum* 62(3)**,** 465-471.

Bretzloff Jr, C.W. (1954). The growth and fruiting of Sordaria fimicola. *American Journal of Botany* 41**,** 58-67.

Brown, T.S., and Merrill, W. (1973). Germination of basidiospores of Fomes applanatus. *Phytopathology* 63**,** 547-550.

Brown, W. (1922). Studies in the physiology of parasitism. IX. The effect on the germination of fungal spores of volatile substances arising from plant tissues. *Annals of Botany* 36(143)**,** 285-300.

Bukowski, T. (1954). The influence of ethylene-bichloride, C2H4Cl2 on the germination of the spores of Agaricus hortensis cooke. *Acta Microbiologica Polonica* 3(4).

Buston, H., Moss, M., and Tyrrell, D. (1966). The influence of carbon dioxide on growth and sporulation of Chaetomium globosum. *Transactions of the British Mycological Society* 49(3)**,** 387-IN386.

Currie, C.R., Scott, J.A., Summerbell, R.C., and Malloch, D. (1999). Fungus-growing ants use antibiotic-producing bacteria to control garden parasites. *Nature* 398(6729)**,** 701-704.

Deveau, A., Brulé, C., Palin, B., Champmartin, D., Rubini, P., Garbaye, J., et al. (2010). Role of fungal trehalose and bacterial thiamine in the improved survival and growth of the ectomycorrhizal fungus Laccaria bicolor S238N and the helper bacterium Pseudomonas fluorescens BBc6R8. *Environmental microbiology reports* 2(4)**,** 560-568.

Dijkstra, F.I., Scheffers, W., and Wiken, T. (1972). Submerged growth of the cultivated mushroom, Agaricus bisporus. *Antonie van Leeuwenhoek* 38(1)**,** 329-340.

Duggar, B.M. (1901). Physiological studies with reference to the germination of certain fungous spores. *Botanical Gazette* 31(1)**,** 38-66.

Duponnois, R., and Garbaye, J. (1990). Some mechanisms involved in growth stimulation of ectomycorrhizal fungi by bacteria. *Canadian Journal of Botany* 68(10)**,** 2148-2152.

Ellis, J., and Hesseltine, C. (1962). Rhopalomyces and Spinellus in pure culture and the parasitism of Rhopalomyces on nematode eggs. *Nature* 193(4816)**,** 699-700.

Emerson, M.R. (1948). Chemical activation of ascospore germination in Neurospora crassa. *Journal of bacteriology* 55(3)**,** 327-330.

Emerson, M.R. (1954). Some physiological characteristics of ascospore activation in Neurospora crassa. *Plant physiology* 29(5)**,** 418-428.

Falck, R. (1912). "Die Meruliusfäule des Bauholzes," in *Haus-schwammforschungen. Heft 6,* ed. A. Möller. (Jena: Fischer), 405.

Farkas, G., and Ledingham, G. (1959). The relation of self-inhibition of germination to the oxidative metabolism of stem rust uredospores. *Canadian journal of microbiology* 5(2)**,** 141-151.

Ferguson, M. (1902). A preliminary study of the germination of the spores of Agaricus campestris and other Basidiomycetous fungi. *U.S. Department of Agriculture Bureau of Plant Industry Bulletin* 16.

Fletcher, J., and Morton, A. (1970). Physiology of germination of Penicillium griseofulvum conidia. *Transactions of the British Mycological Society* 54(1)**,** 65-81.

French, R., and Weintraub, R. (1957). Pelargonaldehyde as an endogenous germination stimulator of wheat rust spores. *Archives of biochemistry and biophysics* 72(1)**,** 235-237.

French, R.C. (1961). Stimulation of uredospore germination in wheat stem rust by terpenes and related compounds. *Botanical Gazette* 122(3)**,** 194-198.

Fries, N. (1942). Über die Sporenkeimung bei einigen Gasteromyceten und mykorrhizabildenden Hymenomyceten. *Archiv für Mikrobiologie* 12(1)**,** 266-284.

Fries, N. (1949). Culture studies in the genus Mycena. *Svensk botanisk Tidskrift* 43**,** 316-342.

Fries, N. (1966). Chemical factors in the germination of spores of Basidiomycetes. *The Fungus Spores*.

Fries, N. (1976). Spore germination in Boletus induced by amino acids. *Proceedings of the Koninklijke Nederlandse Akademie van Wetenschappen, Series C. Biological and medical sciences* 79**,** 142-146.

Fries, N. (1978). Basidiospore germination in some mycorrhiza-forming Hymenomycetes. *Transactions of the British Mycological Society* 70(3)**,** 319-324.

Fries, N. (1985). "Spore germination in ectomycorrhizal fungi", in: *6th North American Conference on Mycorrhizae, Bend, Oregon (USA), 25-29 Jun 1984*: Oregon State University. Forest Research Laboratory).

Fries, N. (1989). The influence of tree roots on spore germination of ectomycorrhizal fungi. *Agriculture, Ecosystems & Environment* 28(1-4)**,** 139-144.

Fries, N., and Birraux, D. (1980). Spore germination in Hebeloma stimulated by living plant roots. *Experientia* 36(9)**,** 1056-1057.

Fries, N., Serck-Hanssen, K., Dimberg, L.H., and Theander, O. (1987). Abietic acid, and activator of basidiospore germination in ectomycorrhizal species of the genus Suillus (Boletaceae). *Experimental Mycology* 11(4)**,** 360-363.

Fries, N., and Swedjemark, G. (1986). "Specific effects of tree roots on spore germination in the ectomycorrhizal fungus, Hebeloma mesophaeum (Agaricales)", in: *Physiological and Genetical Aspects of Mycorrhizae. Aspects physiologiques et genetiques des mycorhizes. Dijon (France). 1-5 Jul 1985.*).

Gassner, G., and Niemann, E. (1955). Über die Beeinflussung der Sporenkeimung des Zwergsteinbrandes und Roggensteinbrandes durch verschiedene Chemikalien. *Phytopathologische Zeitschrift* 23**,** 121-140.

Goddard, D.R. (1935). The reversible heat activation inducing germination and increased respiration in the ascospores of Neurospora tetrasperma. *The Journal of general physiology* 19(1)**,** 45-60.

Haskins, R.H., and Weston Jr, W.H. (1950). Studies in the lower Chytridiales. I. Factors affecting pigmentation, growth, and metabolism of a strain of Karlingia (Rhizophlyctis) rosea. *American Journal of Botany***,** 739-750.

Heilmann-Clausen, J., and Boddy, L. (2005). Inhibition and stimulation effects in communities of wood decay fungi: exudates from colonized wood influence growth by other species. *Microbial ecology* 49(3)**,** 399-406.

Herrera-Martínez, A., Ruiz-Medrano, R., Galván-Gordillo, S.V., Toscano-Morales, R., Gómez-Silva, L., Valdés, M., et al. (2014). A 2-component system is involved in the early stages of the Pisolithus tinctorius-Pinus greggii symbiosis. *Plant signaling & behavior* 9(5)**,** e28604.

Hildebrandt, U., Janetta, K., and Bothe, H. (2002). Towards growth of arbuscular mycorrhizal fungi independent of a plant host. *Applied and environmental microbiology* 68(4)**,** 1919-1924.

Hildebrandt, U., Ouziad, F., Marner, F.-J., and Bothe, H. (2006). The bacterium Paenibacillus validus stimulates growth of the arbuscular mycorrhizal fungus Glomus intraradices up to the formation of fertile spores. *FEMS Microbiology Letters* 254(2)**,** 258-267.

Hintikka, V. (1970). Stimulation of spore germination of wood-decomposing Hymenomycetes by carbon dioxide. *Karstenia* 11**,** 23-27.

Horowitz, N., Charlang, G., Horn, G., and Williams, N.P. (1976). Isolation and identification of the conidial germination factor of Neurospora crassa. *Journal of Bacteriology* 127(1)**,** 135-140.

Kameoka, H., Tsutsui, I., Saito, K., Kikuchi, Y., Handa, Y., Ezawa, T., et al. (2019). Stimulation of asymbiotic sporulation in arbuscular mycorrhizal fungi by fatty acids. *Nature microbiology* 4(10)**,** 1654-1660.

Kehl, H. (1943). Zur Keimungsphysiologie der Champignonsporen. *Die Gartenbauwissenschaft* 17(2)**,** 156-170.

Kikuchi, K., Matsushita, N., Suzuki, K., and Hogetsu, T. (2007). Flavonoids induce germination of basidiospores of the ectomycorrhizal fungus Suillus bovinus. *Mycorrhiza* 17(7)**,** 563-570. doi: 10.1007/s00572-007-0131-8.

Krishna, A., and Sharma, B. (1987). "Studies on Spcre Germination CF Pholiota Destruens (Brond) Gillet," in *Developments in crop science*. Elsevier), 69-75.

Kögl, F., and Fries, N. (1937). Über den Einfluß von Biotin, Aneurin und Meso-Inosit auf das Wachstum verschiedener Pilzarten. *Zeitschrift für physiologische Chemie* 249(2-4)**,** 93-110.

Lagrange, H., Jay‐Allgmand, C., and Lapeyrie, F. (2001). Rutin, the phenolglycoside from eucalyptus root exudates, stimulates Pisolithus hyphal growth at picomolar concentrations. *New Phytologist* 149(2)**,** 349-355.

Laiho, O. (1970). Paxillus involutus as a mycorrhizal symbiont of forest trees. *Acta Forestalia Fennica* 106.

Lehrian, D.W., Schisler, L.C., and Patton, S. (1976). The effects of linoleate and acetate on the growth and lipid composition of mycelium of Agaricus bisporus. *Mycologia* 68(3)**,** 453-462.

Leszczenko, P. (1928). Studies of the actions of solutions of salts, alkalis, and acids on the spores of some pathogenic fungi. *Prace Wydzialu chorób i szkodników róslin Państwowego instytutu naukowego gospodarstva wiejskiego w Bydgoszczy* 6**,** 1-37.

Ling, L. (1940). Factors affecting spore germination and growth of Urocystis occulta in culture. *Phytopathology* 30(7)**,** 579-591.

Lösel, D.M. (1964). The stimulation of spore germination in Agaricus bisporus by living mycelium. *Annals of Botany* 28(4)**,** 541-554.

Machlis, L., and Ossia, E. (1953). Maturation on the meiosporangia of Euallomyces. I. The effect of cultural conditions. *American Journal of Botany***,** 358-365.

Maier, A., Riedlinger, J., Fiedler, H.-P., and Hampp, R. (2004). Actinomycetales bacteria from a spruce stand: characterization and effects on growth of root symbiotic and plant parasitic soil fungi in dual culture. *Mycological Progress* 3(2)**,** 129-136.

Maneval, W. (1922). Germination of teliospores of rusts at Columbia, Missouri. *Phytopathology* 12**,** 471-488.

Marx, D.H., and Ross, E.W. (1970). Aseptic synthesis of ectomycorrhizae on Pinus taeda by basidiospores of Thelephora terrestris. *Canadian Journal of Botany* 48(1)**,** 197-198.

Mayo, K., Davis, R.E., and Motta, J. (1986). Stimulation of germination of spores of Glomus versiforme by spore-associated bacteria. *Mycologia* 78(3)**,** 426-431.

McCracken, F.I. (1982). Some factors affecting basidiospore germination of Pleurotus sapidus. *Canadian Journal of Botany* 60(9)**,** 1658-1661.

McTeague, D., Hutchinson, S., and Reed, R. (1959). Spore germination in Agaricus campestris L. ex Fr. *Nature* 183(4677)**,** 1736-1736.

Melin, E. (1962). Physiological aspects of mycorrhizae of forest trees. *Tree growth***,** 247-263.

Melin, E., and Rama Das, V. (1954). Influence of root‐metabolites on the growth of tree mycorrhizal fungi. *Physiologia Plantarum* 7(4)**,** 851-858.

Mosse, B. (1959). The regular germination of resting spores and some observations on the growth requirements of an Endogone sp. causing vesicular-arbuscular mycorrhiza. *Transactions of the british mycological society* 42(3)**,** 273-IN274.

Niemann, E. (1956). Stimulationswirkung von Düngemitteln und quecksilberhaltigen Beizmitteln auf die Sporenkeimung des Zwergsteinbrandes (Tilletia contraversa Kühn). *Angewandte Botanik* 30**,** 1-13.

Niemann, E. (1957). Stimulationswirkung von Quecksilbeverbindungen auf die Sporenkeimung des Zwergsteinbrandes. *Angewandte Botanik* 31**,** 191-196.

Noble, R. (1924). Studies on the parasitism of Urocystis tritici Koern., the organism causing flag smut of wheat. *Journal of agricultural Research* 27**,** 451-489.

Oort, A.J.P. (1974). Activation of spore germination in Lactarius species by volatile compounds of Ceratocystis fagacearum. *Proceedings of the Koninklijke Nederlandse Akademie van Wetenschappen, Series C* 77(4)**,** 301-307.

Page, D.E., Glen, M., Ratkowsky, D.A., Beadle, C.L., Rimbawanto, A., and Mohammed, C.L. (2017). Ganoderma basidiospore germination responses as affected by spore density, temperature and nutrient media. *Tropical Plant Pathology* 42(5)**,** 328-338.

Platz, G. (1928). The relation of oxygen to the germination of the chlamydospores of Ustilago zeae (Beckm.) Ung. Iowa State College Journal of Science 2**,** 137-143.

Rabinovitz-Sereni, D. (1931). Perdita della facoltà germinativa delle spore di Deuterophoma tracheiphila alla fine del periodo primaverile. *Boll. R. Stazione Pat,. Veg. Roma* 11**,** 154-157.

Riedlinger, J., Schrey, S.D., Tarkka, M.T., Hampp, R., Kapur, M., and Fiedler, H.-P. (2006). Auxofuran, a novel metabolite that stimulates the growth of fly agaric, is produced by the mycorrhiza helper bacterium Streptomyces strain AcH 505. *Applied and environmental microbiology* 72(5)**,** 3550-3557.

Rikhvanov, E.G., Varakina, N.N., Sozinov, D.Y., and Voinikov, V.K. (1999). Association of bacteria and yeasts in hot springs. *Applied and Environmental Microbiology* 65(9)**,** 4292-4293.

Rippel, A., and Bortels, H. (1927). Vorläufige Versuche über die allgemeine Bedeutung der Kohlensäure für die Pflanzenzelle (Versuche an Aspergillus niger). *Biochemische Zeitschrift* 184**,** 237-244.

Ruan, Y., Kotraiah, V., and Straney, D.C. (1995). Flavonoids stimulate spore germination in Fusarium solani pathogenic on legumes in a manner sensitive to inhibitors of cAMP-dependent protein kinase. *MPMI-Molecular Plant Microbe Interactions* 8(6)**,** 929-938.

Sbaraini, N., Hu, J., Roux, I., Phan, C.-S., Motta, H., Rezaee, H., et al. (2021). Polyketides produced by the entomopathogenic fungus Metarhizium anisopliae induce Candida albicans growth. *Fungal Genetics and Biology***,** 103568.

Schaffnit, E. (1926). Zur Physiologie von Ustilago hordei Kell. u. Sw. *Berichte der Deutschen Botanischen Gesellschaft* 44**,** 151-156.

Schisler, L.C., and Volkoff, O. (1977). The effect of safflower oil on mycelial growth of Boletaceae in submerged liquid cultures. *Mycologia* 69(1)**,** 118-125.

Schopfer, W. (1942). Les facteurs de croissance pour rhizopus suinus. Déterminisme et relativité des pouvoirs de synthése. *Actes de la Société helvétique des sciences naturelles***,** 122-123.

Schrey, S.D., Salo, V., Raudaskoski, M., Hampp, R., Nehls, U., and Tarkka, M.T. (2007). Interaction with mycorrhiza helper bacterium Streptomyces sp. AcH 505 modifies organisation of actin cytoskeleton in the ectomycorrhizal fungus Amanita muscaria (fly agaric). *Current genetics* 52(2)**,** 77-85.

Sibilia, C. (1930). Researches on cereal rusts. II. The germination of the teleutospores of Puccinia graminis and P. triticina. *Bollettino della R. Stazione di Patologia Vegetale* 10(2)**,** 164-190.

Stack, R., Sinclair, W., and Larsen, A. (1975). Preservation of basidiospores of Laccaria laccata for use as mycorrhizal inoculum. *Mycologia* 67(1)**,** 167-170.

Stakman, E., Cassell, R., and Moore, M. (1934). The cytology of Urocystis occulta. *Phytopathology* 24(8)**,** 874-889.

Sumere, C.V., Preter, C.V.S.-D., and Ledingham, G. (1957). Cell-wall-splitting enzymes of Puccinia graminis var. tritici. *Canadian journal of microbiology* 3(5)**,** 761-770.

Sun, Y.-P., and Fries, N. (1992). The effect of tree-root exudates on the growth rate of ectomycorrhizal and saprotrophic fungi. *Mycorrhiza* 1(2)**,** 63-69.

Sussman, A.S. (1953). The effect of furfural upon the germination and respiration of ascospores of Neurospora tetrasperma. *American Journal of Botany* 40**,** 401-404.

Thiel, A., and Weiss, F. (1920). The effect of citric acid on the germination of the teliospores of Puccinia graminis tritici. *Phytopathology* 10**,** 448-452.

Tillotson, R., Wösten, H., Richter, M., and Willey, J. (1998). A surface active protein involved in aerial hyphae formation in the filamentous fungus Schizophillum commune restores the capacity of a bald mutant of the filamentous bacterium Streptomyces coelicolor to erect aerial structures. *Molecular microbiology* 30(3)**,** 595-602.

Turel, F.L. (1955). Influence of methyl-p-hydroxybenzoate, chlortetracycline, and certain trace metals on germination of uredospores of Melampsora occidentalis Jacks. *Canadian journal of microbiology* 1(5)**,** 293-298.

Tylutki, E.E. (1955). *A Study of Some Aspects of Morphology, Genetics, and Cultural Behavior of the Heterothallic Pyrenomycete Gelasinospora Calospora (Mouton) Moreau Et Moreau, Var. Autosteira (Alexopoulos Et Sun) Alexopoulos Et Sun.* Doctoral dissertataion. Michigan State University of Agriculture and Applied Science, East Lansing.

Uppal, B. (1924). Spore germination of Phytophthora infestans. *Phytopathology* 14**,** 32-33.

Uppal, B. (1926). Relation of oxygen to spore germination in some species of the Peronosporales. *Phytopathology* 16**,** 285-292.

von Guttenberg, H., and Strutz, I. (1952). Zur Keimungsphysiologie von Ustilago zeae. *Archiv für Mikrobiologie* 17(1)**,** 189-198.

Wardle, K.S., and Schisler, L.C. (1969). The effects of various lipids on growth of mycelium of Agaricus bisporus. *Mycologia* 61(2)**,** 305-314.

Watson, P. (1964). Spore germination in Spinellus macrocarpus. *Transactions of the British Mycological Society* 47(2)**,** 239-IN234.

Welsford, E. (1907). Fertilization in Ascobolus furfuraceus, Pers. *New Phytologist* 6(6)**,** 156-161.

Whaley, J.W., and Barnett, H. (1963). Parasitism and nutrition of Gonatobotrys simplex. *Mycologia* 55(2)**,** 199-210.

Wilhelm, P. (1931). Studien zur Spezialisierungsweise des Weizengelbrostes, Puccinia glumarum f. sp. tritici (Schmidt) Erikss. et Henn. und zur Keimungsphysiologie seiner Uredosporen. *Arb. Biol. Reichsanst. Land- u. Forstwirtsch.* 19**,** 1-23.

Wood-Baker, A. (1955). Effects of oxygen-nitrogen mixtures on the spore germination of mucoraceous moulds. *Transactions of the British Mycological Society* 38(3)**,** 291-297.

Xu, X.-L., Lee, R.T.H., Fang, H.-M., Wang, Y.-M., Li, R., Zou, H., et al. (2008). Bacterial peptidoglycan triggers Candida albicans hyphal growth by directly activating the adenylyl cyclase Cyr1p. *Cell host & microbe* 4(1)**,** 28-39.

Yanagita, T. (1957). Biochemical aspects on the germination of conidiospores of Aspergillus niger. *Archiv für Mikrobiologie* 26(4)**,** 329-344.

Yates, A., Seaman, A., and Woodbine, M. (1968). Ascospore germination in Byssochlamys nivea. *Canadian journal of microbiology* 14(4)**,** 319-325.

Yu, C.C.-C. (1954). The culture and spore germination of Ascobolus with emphasis on A. magnificus. *American Journal of Botany* 41**,** 21-30.

Zeng, R.S., Mallik, A.U., and Setliff, E. (2003). Growth stimulation of ectomycorrhizal fungi by root exudates of Brassicaceae plants: role of degraded compounds of indole glucosinolates. *Journal of chemical ecology* 29(6)**,** 1337-1355.
